# Articaine in functional NLC show improved anesthesia and anti-inflammatory activity in zebrafish

**DOI:** 10.1038/s41598-020-76751-6

**Published:** 2020-11-12

**Authors:** Gustavo H. Rodrigues da Silva, Gabriela Geronimo, Juan P. García-López, Lígia N. M. Ribeiro, Ludmilla D. de Moura, Márcia C. Breitkreitz, Carmen G. Feijóo, Eneida de Paula

**Affiliations:** 1 Department of Biochemistry and Tissue Biology, Institute of Biology, University of Campinas-UNICAMP, Rua Monteiro Lobato, 255, Cid. Universitária Zeferino Vaz, Campinas, São Paulo, 13083862 Brazil; 2 Laboratory of Fish Immunology, Department of Biological Sciences, Faculty of Life Sciences, Andres Bello University, 8370146 Santiago, Chile; 3 Department of Analytical Chemistry, Institute of Chemistry, UNICAMP, Campinas, São Paulo, Brazil

**Keywords:** Dentistry, Drug delivery, Drug delivery

## Abstract

Anesthetic failure is common in dental inflammation processes, even when modern agents, such as articaine, are used. Nanostructured lipid carriers (NLC) are systems with the potential to improve anesthetic efficacy, in which active excipients can provide desirable properties, such as anti-inflammatory. Coupling factorial design (FD) for in vitro formulation development with in vivo zebrafish tests, six different NLC formulations, composed of synthetic (cetyl palmitate/triglycerides) or natural (avocado butter/olive oil/copaiba oil) lipids were evaluated for loading articaine. The formulations selected by FD were physicochemically characterized, tested for shelf stability and in vitro release kinetics and had their in vivo effect (anti-inflammatory and anesthetic effect) screened in zebrafish. The optimized NLC formulation composed of avocado butter, copaiba oil, Tween 80 and 2% articaine showed adequate physicochemical properties (size = 217.7 ± 0.8 nm, PDI = 0.174 ± 0.004, zeta potential = − 40.2 ± 1.1 mV, %EE = 70.6 ± 1.8) and exhibited anti-inflammatory activity. The anesthetic effect on touch reaction and heart rate of zebrafish was improved to 100 and 60%, respectively, in comparison to free articaine. The combined FD/zebrafish approach was very effective to reveal the best articaine-in-NLC formulation, aiming the control of pain at inflamed tissues.

## Introduction

Local anesthetics (LA) are essential drugs in dental practice, used to prevent pain by blockage of peripheral nerves. Inflammatory processes, as in cases of pulpitis and periodontitis, provoke alterations in dental tissues during treatments and contribute to anesthesia failure (which rates may reach 70%)^1^. Other outcomes such as increased blood flow, local pH change (acidification) and increased nerve sensitization, contribute to anesthesia failure in inflamed tissues^1^. In this scenario, it is necessary to increase the LA concentration to attain the desired antinociceptive action. However, higher amounts of LA increase the risk of systemic toxicity, limiting the total administered dose^2^. Articaine (ATC), released on the market in 1984, is the only aminoamide local anesthetic with a thiophene ring as the lipophilic moiety^3^. Its use became popular in dentistry^4^ with reports of slightly better anesthesia in cases of pulp inflammation^5^. Even though higher doses of articaine can be administered—because of its low half-life—than other LA agents^4^, this is not enough to prevent anesthetic failure in inflamed tissues. Thus, new strategies are needed to increase ATC efficiency without causing adverse effects on the patients.

Lipid-based drug delivery systems have been shown useful to increase the therapeutic activity of LA, providing sustained release, extended action and decreased toxicity^6^. Nanostructured lipid carriers (NLC) are drug delivery systems that contain an inner core composed of solid and liquid lipids, stabilized by surfactants^7^. Currently several reports show that NLC can be prepared with natural (active) excipients that may confer functional properties such as anti-inflammatory, anti-microbial, and analgesic^8,9^ to these nanoparticles.

Avocado (*Persia gratissima*) butter, olive (*Olea europaea*) and copaiba (*Copaifera l*.) oils are natural excipients with anti-inflammatory properties. The unsaturated fatty alcohols persenone A and B found in avocado butter have a potent anti-inflammatory activity^10^. Likewise, olive oil possesses oleocanthal, which anti-inflammatory properties are comparable to those of ibuprofen^11^. In the case of copaiba oil, one of its major components, β-caryophyllene, exhibits anti-inflammatory effects^12^. In lipid-based drug delivery systems, olive and copaiba oils have already been tested as excipients that act synergistically with the encapsulated drug or aggregate therapeutic properties to the system^13^. In this way, the use of natural compounds as NLC-excipients arises as a plausible strategy for encapsulating ATC, coupling anesthesia and anti-inflammatory activities in dental treatments under inflammatory conditions.

The premise of testing several excipients creates a large number of possible NLC compositions that can be optimized using factorial design^14^. However, such diversity turns it difficult to screen the formulations with desired biological effects using traditional (rodent) in vivo models, that require a large number of animals and a great deal of time and costs. Therefore, an alternative model to rodents, which allows the analysis of a high number of conditions is desirable^15^. In this scenario, zebrafish (*Danio rerio*) with its large numbers of eggs per brood (approximately 200 eggs/clutch) turns it possible to screen the several produced NLC formulations, at the same time. Other distinctive feature of zebrafish is the optical transparency of its embryos and larvae that, coupled to the several transgenic lines with fluorescent-labelled cell lines available, allows monitoring selected cell types within the whole organism^16^. Of note, zebrafish has interesting similarities to mammals: *ca.* 70% of its genes are similar to mammals, while 47% of human genes have a zebrafish counterpart^17^. Additionally, its immune system is highly conserved in comparison to mammals, at molecular and cellular levels^18^. For these reasons, zebrafish is an established model for the study of inflammatory diseases^19^ and has been used to model several human pathologies^20,21^. These features justify the use of zebrafish as a model for pharmaceutical technology and development^22^.

In this work, we show the development and screening of six NLC formulations loaded with ATC aiming its future use on inflamed tissues to promote better anesthesia with some anti-inflammatory effect. The approach at this development phase consisted in using factorial design coupled with zebrafish model, to select the formulation with the best physicochemical and biological properties. The optimized formulations were characterized regarding number, size, polydispersity and zeta potential of the nanoparticles, encapsulation efficiency and in vitro release of articaine, and shelf stability for 12 months at room temperature. Zebrafish larvae were used as a versatile platform for screening responses in vivo (anti-inflammatory effect of excipients and anesthetic effect of ATC). This simple and straightforward strategy provided data on multiple biological effects for optimization of the developed drug delivery systems, quickly and economically, and already in the initial phase of development.

## Materials and methods

### Materials

Articaine hydrochloride (ATC) was donated by DFL Ind. Com. S.A. (Rio de Janeiro, Brazil). Cetyl palmitate (CP, cat 501427) and Dhaykol 6040 LW (DK, cat SO1253) (caprylic/capric acid triglycerides) were purchased from Dhaymers Química Fina (São Paulo, Brazil). Pluronic F68 (P68, cat K4894), Tween 80 (T80, cat P1754), copaiba oil (CO, cat W521809), methyl cellulose (cat M0512) and ethyl 3-aminobenzoate methanesulfonate (Tricaine, cat E10521) were supplied by Sigma–Aldrich (St. Louis, MO, USA). Avocado butter and olive oil were purchased from Engenharia das Essências Ltda (São Paulo, Brazil). Liss rhodamine-PE (1,2-dipalmitoyl-sn-glycero-3-phosphoethanolamine-*N*-(lissamine rhodamine B sulfonyl)) was from Avanti Polar Lipids, Inc. (Alabaster, AL, USA, cat 810150). Low melting point agarose was obtained from Cleaver Scientific Ltd (Warwickshire, UK, cat CSL-LMA5). E3 medium (5 mM NaCl, 0.17 mM KCl, 0.33 mM CaCl_2_; 0.3 mM MgCl_2_, calibrated to pH 7 with NaHCO_3_) was prepared at the Fish Immunology lab, Andres Bello University. Deionized water (18 MΩ) was obtained with an Elga USF Maxima ultra-pure water purifier.

### Formulation preparation

NLCs were prepared using the emulsification-ultrasonication method^23^. Briefly, the mixture of lipids and ATC in base form was melted (10 °C above the melting point of the solid lipid) in a water bath. A solution of surfactant was heated to the same temperature and both phases were blended under high-speed agitation (10,000 rpm), for 3 min in an Ultra-Turrax blender (IKA Werke, Staufen, Germany). After, the mixture was sonicated for 15 min in a Vibracell tip sonicator (Sonics & Mat. Inc., Danbury, USA) operated at 500 W and 20 kHz, in alternating 30 s (on/off) cycles. To form the NLC, the resultant nanoemulsion was immediately cooled to room temperature, with an ice bath.

### Characterization of formulations

#### Physicochemical characterization

A Nano ZS90 analyzer (Malvern Instruments, UK) was used to determine the nanoparticles hydrodynamic size (size) and polydispersity index (PDI) by measuring the intensity of the scattered light. Zeta potential (ZP, millivolts) were measured by laser doppler microelectrophoresis, with the same equipment. Nanoparticle concentration (NC, expressed in number of particles/mL) was obtained with a NS300 NTA instrument (NanoSight, Amesbury, UK) equipped with a 532 nm laser. In all cases the samples were diluted in deionized water (1000–50,000x, n = 3).

#### Transmission electron microscopy (TEM)

The NLC formulations (10 μL) were added to a carbon grid (200 Mesh, copper). After 2 min, 10 μL of a 2% (w/w) aqueous solution of uranyl acetate was added on top of it, for negative contrast. After 1 min, the excess volume was removed, and 10 μL of deionized water was added to the sample. The prepared grid remained at rest for 24 h, at room temperature, for drying. The electron micrographs were performed in a LEO 906 Zeiss transmission electron microscope (Carl Zeiss, Germany) operating at 60 kV.

#### Articaine quantification and encapsulation efficiency

A Waters Breeze 2 (Waters Technol., São Paulo, Brazil) high performance liquid chromatography (HPLC) equipment was used, with a Gemini 5 µm, C18, 110 Å, with 150 × 4.6 mm (Phenomenex, Torrance, USA) column at 40 °C. The mobile phase was a mixture with acetonitrile: 50 mM KH_2_PO_4_, 25:75 (v/v), in a 1 mL/min flux. The injection volume was 30 µL and the absorbance was followed at 273 nm^24^. The limits of detection and quantification were, respectively: 0.58 and 1.93 µg/mL. The total amount of ATC (ATC_total_) in the formulations was determined by diluting the samples in the mobile phase (n = 3; 40×)^25^. The encapsulation efficiency (%EE) was measured by the ultrafiltration-centrifugation method, using (30 kDa, Millipore) cellulose filters. The concentration of ATC in the filtrate (ATC_free_) was quantified and the encapsulation efficiency (%EE) was calculated according to Eq. (1)^23^:

1 $$\% EE = \frac{{ATC_{{total{\kern 1pt} {\kern 1pt}^{{ - {\kern 1pt} \mathop {}\nolimits^{{}} }} }} ATC_{free} }}{{ATC_{total} }}x100$$

The amount of articaine loaded in NLCs was also expressed in terms of drug loading capacity, accordingly to Eq. (2)^26^:

2 $$\% Drug\,loading= \frac{weight\,of\,encapsulated\,ATC}{weight\,of\,nanoparticles} x 100$$

### Factorial design

For the six lipid mixtures selected (Table 1), a 2^3^ experimental design with central points in triplicate was performed. The Design Expert software (version 11, Stat-Ease Inc., USA) was used for the analysis of results. Models were considered significant if p < 0.05^14^. The variables and levels are listed in Table 2 and the analyzed responses were: size, PDI and zeta potentials.

**Table 1:** NLC formulations and their constituents.

Type	Abbreviation	Solid lipid	Liquid lipid	Surfactant
Synthetic	NLC-CP1-A	Cetyl Palmitate	Dhaykol 6040	Pluronic F-68
	NLC-CP2-A	Cetyl Palmitate	Dhaykol 6040	Tween 80
Functional	NLC-CO1-A	Avocado Butter	Copaiba oil	Pluronic F-68
	NLC-CO2-A	Avocado Butter	Copaiba oil	Tween 80
	NLC-OO1-A	Avocado Butter	Olive oil	Pluronic F-68
	NLC-OO2-A	Avocado Butter	Olive oil	Tween 80

**Table 2:** Experimental variables and levels used in the 2^3^ experimental design of articaine-in-nanostructured lipid carriers.

Variable	Low Level	High Level
Total lipids (% w/w)	9	15
Surfactant (% w/w)	2.5	5
Articaine (% w/w)	0	4

### In vitro release experiment

The in vitro kinetics of ATC release was measured in a 12 mL Franz diffusion cell system, with 5 mM PBS buffer (pH 7.4, 37 °C) as the receptor solution, separated by a cellulose membrane (Spectra/Por, MWCO 12–14 kDa) from the sample compartment (n = 6). The formulations tested (50 µL) were: 2% (w/w) aqueous solution of articaine hydrochloride (free ATC) and articaine in NLCs (NLC-CP1-A, NLC-CP-2-A, NLC-CO1-A, NLC-CO2-A, NLC-OO1-A and NLC-OO2-A). At determined time intervals (0.15, 0.3, 1, 2, 4, 6, 8, 22, 24, 28 h) 200 µL aliquots were collected from the sampling port and replaced with the PBS buffer, to preserve the sink condition. The amount of ATC in the receptor compartment was quantified by HPLC. The KinetDS 3.0 software was used for the quantitative analysis of the obtained release curves^27^. From several kinetic models tested and according to the R^2^ coefficient, the Weibull model^28^ exhibited the best fit for all NLC formulations (Eq. 3):

3 $$m=1-exp\left[\frac{-{t}^{n}}{a}\right]$$

where *m* is the amount of ATC released as a function of time (*t*), *a* denotes a scale parameter describing the time dependence, and n is the shape parameter of the curve.

### Physical stability study

The physical stability of NLC formulations was monitored during 12 months, at room temperature (25 °C ± 2 °C) and 60 ± 5% humidity^25,29^. The analyzed parameters were nanoparticle size (nm), PDI and ZP (mV). Analysis of variance (ANOVA, 95% confidence level) and Tukey post hoc test were used to compare inter-groups significant differences regarding the initial time measurements with the GraphPad Prism software, version 6.01 (California, USA).

### In vivo Zebrafish tests

#### Zebrafish strains and maintenance

Zebrafish maintenance and experimental protocols were approved by the Animal Ethic Committee of Universidad Andres Bello (certificate 007/2016) and all experiments were carried out complying with guidelines for the use of fishes in research. Homozygous *Tg(BACmpx:GFP)*^*i114*30^ and *TgBAC(cldn15la:GFP)*^*pd1034*31^ fish were used in this work. Adult fish were maintained and raised according to standard protocols^32^ with 14/10 h light/dark cycles and fed with a dry flake food (Gemma micron 300, Skretting, USA), twice a day. All embryos were collected through natural spawning and raised at 28˚C in petri dishes containing E3 medium^32^ (under 14/10 h light/dark cycles). All experiments were performed using 5 days post fertilization (dpf) larvae.

#### NLC absorption in zebrafish larvae

Eight *TgBAC(cldn15la:GFP)*^pd1034^ larvae, were incubated in 6 well plates containing 2 mL of E3 medium with the NLCs (10^10^ nanoparticle/mL) loaded with 0.01% w/w Liss rhodamine-PE (%EE > 99%; Supplementary Table S7) with and without ATC for either 1 h; 12 h or 24 h. After incubation, larvae were washed with E3 medium, anesthetized with 0.017% tricaine, and mounted in 4% methylcellulose or 1% low melting point agarose to be analyzed under a fluorescence stereomicroscope or a confocal microscope, using RFP (Red-fluorescence) and GFP (Green-fluorescence) filters.

#### Anti-inflammatory activity of NLC excipients

Eight *Tg(BACmpx:GFP)*^*i114*^ larvae were selected with similar amount of neutrophils in the caudal hematopoietic tissue and less than 5 neutrophils in the somite region^33^. After, selected larvae were incubated in E3 medium alone or containing NLCs (10^12^ nanoparticles/mL) formulations without ATC for 1 h. After incubation, larvae were washed, anesthetized with 0.017% tricaine and the caudal fin was amputated with a microsurgical knife (World Precision Instruments, UK, cat 500249) to induce an acute inflammatory process, as described by Elks and collaborators^34^. At 3 h post damage (hpd), when the maximum number of neutrophils was present at the injury, their amount at the damaged area (size: 200 µm long and 30 µm width) was quantified.

#### Bradycardia and bradycardia recovery

Eight *Tg(BACmpx:GFP)*^*i114*^ larvae were incubated in E3 medium, with free ATC (0.5 and 1 mM), NLCs loaded with ATC (0.5 or 1.0 mM), or NLCs without ATC (10^12^ nanoparticles/mL), for 1 h. Then, the larvae heartbeats were quantified for 60 s under a stereomicroscope using a manual counter. For bradycardia recovery, larvae were treated with 1 mM ATC (free and in NLCs-ATC), or NLCs without ATC (10^12^ nanoparticles/mL) for 1 h. After, the solution was washed, and heartbeats were quantified at 1-, 2- and 3-h post removal (hpr) of treatment.

#### Anesthesia effect: touch reaction

Eight *Tg(BACmpx:GFP)*^*i114*^ larvae were incubated in E3 medium, with free ATC (0.5 and 1 mM), NLCs loaded with ATC (0.5 or 1.0 mM), or NLCs without ATC (10^12^ nanoparticles/mL), for 1 h. After incubation, a mechanic stimulus was executed, by touching the larvae with a blunt needle on the head. To measure the larvae response, the following scale was used: score 0 = non-reactive larva, score 1 = larva reaction between 1–3 s, and score 2 = larva reaction in less than 1 s after touch.

#### Zebrafish imaging and statistics

Fluorescent images were captured using either a Leica M205 stereomicroscope with metal-halide lamp (two objectives—0.63× and 1.0×, motorized zoom with 7.8—160 × magnification range) with a DFC 7000 T camera and the software Leica Applications Suite X, v. 3.3 (Leica Microsystems, Germany), or a Leica SP8 confocal laser microscope (with HPL APO CS2 40× oil, numerical aperture of 1.30, and objective lens working distance = 0.24 mm). For image editing ImageJ 1.52a (National Institutes of Health, Maryland, USA. https://imagej.nih.gov/ij/) software was used. For all experiments three biological replicates were executed. Statistical analysis was performed using one-way ANOVA with a post-hoc Tukey test. For all endpoints, p value < 0.05 was considered statistically significant and the GraphPad Prism software, version 6.01 (California, USA) was used. Data were displayed as mean ± standard error.

## Results and discussion

### Factorial design

Pre-formulation tests, in which the selected excipients were formulated with the drug in order to test their physicochemical compatibility^25,35^ were performed (data not shown). From that study we have selected the excipients for articaine encapsulation. Six types of NLCs were proposed: two composed of synthetic lipids and four containing natural active lipids, with either Pluronic F-68 or Tween 80 as surfactants (Table 1).

After assessing articaine compatibility with these excipients, their relative proportions and influence on the properties (size, PDI and ZP) of the nanoparticles were evaluated by factorial design. This tool allowed us, with a reduced number of experiments, to find the conditions that lead to the effective optimization of the system^14^. For each composition, an experimental design was performed following the parameters on Table 2. The complete data, for each experimental design, can be found in the supplementary material (Supplementary Tables S1–S3; Supplementary Fig. S1–S6). Table 3 compiles the results of the experimental designs.

**Table 3:** Compilation of results from the six experimental designs for the encapsulation of articaine in nanostructured lipid carriers.

Formulation	Size (nm) - range of variation	Significant variables for size		PDI - range of variation	Significant variables for PDI		ZP |mV| - range of variation	Significant variables for ZP	
		Pos	Neg		Pos	Neg		Pos	Neg
NLC-CP1-A	182.7–303.2	–	P68	0.160–0.188	–	P68, ATC	31.9–46.3	ATC	–
NLC-CP2-A	237.6–585.0	–	T80	0.095–0.230	T80	TL, ATC	32.1–47.0	ATC	–
NLC-CO1-A	223.7–364.8	TL	P68	0.170–0.329	–	P68, ATC	31.7–49.1	ATC	–
NLC-CO2-A	187.6–282.0	TL	T80, ATC	0.147–0.210	–	ATC	27.5–41.0	ATC	–
NLC-OO1-A	216.8–322.0	ATC	P68	0.160–0.231	ATC	P68	29.3–47.8	ATC	–
NLC-OO2-A	204.6–302.1	TL	T80	0.080–0.244	–	ATC	32.5–51.9	ATC	–

Significant variables with positive (Pos) and negative (Neg) effects. *TL* total lipids, *P68* Pluronic F-68, *T80* Tween 80, *ATC* articaine.

The size of the nanoparticles varied from 182.7–364.8 nm, except for the NLC-CP2-A formulation, which particles reached 585.0 nm. So, the factorial design was driven to choose the nanoparticles with the smallest sizes possible, suitable for parenteral delivery^36^. As expected, surfactants displayed negative effects on nanoparticle size of all formulations, while total lipid concentration (TL) had a positive influence in the size of almost all formulations^25,37^. When analyzing PDI, low values (< 0.25) are preferred because they indicate a narrow size distribution^38^, therefore, as for particle sizes, the goal was to minimize PDI response. Indeed, all formulations but NLC-CO1-A produced particles with size polydispersity below 0.25. As for the ZP, values higher than 20 mV (in modulus) are desirable to assure the colloidal stability of the nanoparticles^39^. Negative ZP values higher than |−27| mV were measured, and ATC displayed a positive effect on the absolute zeta potential values of all formulations.

The desirability criterion was applied (lower size, PDI < 0.25 and ZP > 20 mV in modulus^23^), to simultaneously optimize the three responses for each NLC system and, as shown in Supplementary Figure S7, the central point of different formulations reached a high degree of desirability. Thus, the six chosen formulations were composed of 12.5% total lipids (70:30% solid lipid: liquid lipid), 3.75% surfactant and 2% articaine (w:w). These formulations are shown in Table 4 with their respective controls, prepared without ATC. All the six formulations showed high ATC encapsulation efficiency (> 70%) and drug loading (> 8%) in agreement with previous reports on the encapsulation and drug loading of aminoamide local anesthetics in NLC^25,37^. For these optimized formulations, further in vitro and in vivo tests were carried out.

**Table 4:** Characterization of optimized formulations and their controls (without articaine), regarding average diameters (Size), polydispersity (PDI), zeta potential (ZP), nanoparticles concentration (NC, nanoparticles/mL), encapsulation efficiency (%EE) and drug loading (DL, %).

Formulation	Size (nm)	PDI	ZP (mV)	NC (× 10^13^/mL)	%EE	DL (%)
NLC-CP-A	261.8 ± 3.8	0.178 ± 0.021	− 36.8 ± 0.2	4.68 ± 0.27	73.3 ± 0.2	8.2 ± 0.1
NLC-CP1	230.6 ± 2.5	0.099 ± 0.013	− 31.1 ± 0.7	6.96 ± 0.14	**-**	
NLC-CP2-A	228.9 ± 0.3	0.133 ± 0.036	− 37.9 ± 0.9	5.50 ± 0.40	74.0 ± 0.1	8.3 ± 0.1
NLC-CP2	227.1 ± 2.3	0.184 ± 0.034	− 26.9 ± 0.3	7.30 ± 0.17	**–**	
NLC-CO1-A	269.3 ± 0.4	0.198 ± 0.003	− 42.8 ± 1.7	4.28 ± 0.76	75.8 ± 0.1	8.5 ± 0.1
NLC-CO1	220.9 ± 0.1	0.131 ± 0.012	− 33.0 ± 0.5	4.80 ± 0.52	**–**	
NLC-CO2-A	189.6 ± 1.9	0.140 ± 0.006	− 40.8 ± 0.3	8.14 ± 0.71	78.4 ± 0.1	8.8 ± 0.1
NLC-CO2	206.9 ± 1.0	0.161 ± 0.007	− 23.7 ± 0.3	6.15 ± 0.81	**–**	
NLC-OO1-A	265.0 ± 3.4	0.197 ± 0.031	− 47.5 ± 1.5	5.34 ± 0.80	72.2 ± 0.3	8.1 ± 0.1
NLC-OO1	236.4 ± 0.5	0.163 ± 0.030	− 31.5 ± 0.7	6.08 ± 0.70	**–**	
NLC-OO2-A	230.6 ± 1.9	0.125 ± 0.016	− 43.0 ± 1.5	8.85 ± 0.13	74.9 ± 0.1	8.3 ± 0.1
NLC-OO2	225.7 ± 0.6	0.205 ± 0.022	− 26.5 ± 0.1	4.90 ± 0.21	**–**	

**Figure 1:**
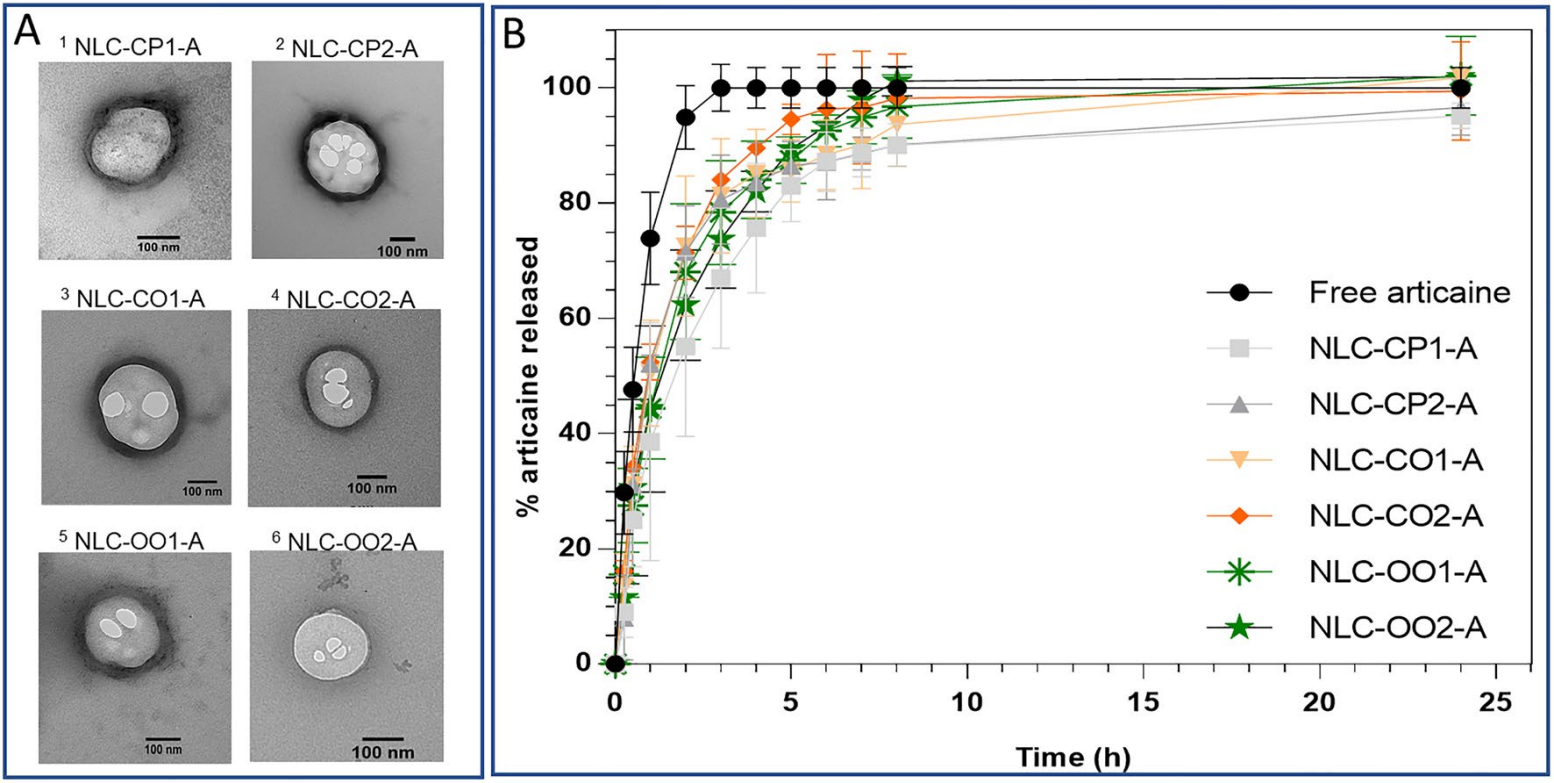
(**A**) Representative TEM micrographs of the nanostructured lipid carriers prepared for articaine; magnification: 100000x (images 2–5), 129,300× (1, 6). (**B**) In vitro release curves of articaine at 37 °C, free (in solution) or encapsulated into the optimized NLC formulations (n = 6). The images in (**A**) were edited with ImageJ software v.1.52a (https://imagej.nih.gov/ij/).

Transmission electron microscopy were carried out to confirm the size and morphology of the nanoparticles^7^. The micrographs (Fig. 1A, Supplementary Fig. S9) revealed spherical nanoparticles with defined borders in the range of 180 nm (NLC-CP1-A) to 320 nm (NLC-CP2-A). These findings confirmed the nanoparticles formation by the used preparation method, and the reliability of DLS measurements, as seen before^25,40^.

### In vitro release of articaine

The in vitro release test provides a suitable measure of the drug release rate from NLC^29^. Figure 1B shows the results of the in vitro release kinetics of ATC, free or encapsulated into each of the six optimized NLC formulations. The equilibrium of articaine in solution (free ATC) was reached in less than 3 h, as previously observed with other hydrophilic local anesthetics such as lidocaine and prilocaine^29^. All the NLC formulations displayed an initial fast release phase, similar to that observed with free ATC, for up to 30% of drug release, followed by a slower release period (3–8 h), when all formulations reached the equilibrium. No significant differences in the release profile of ATC were found among the six NLC formulations, but for those based on cetyl palmitate (NLC-CP1, NLC-CP2) that did not reach 100% release, probably because of prolonged liberation. In the mathematical treatment of the release curves the Weibull model showed the best-fit for all six formulations (Supplementary Table S8). The profile of drug release was compatible with an initial burst phase (until 30%) followed by sustained release of ATC, from the nanoparticles to the water phase. Despite the lack of significant differences among the formulations in relation to the kinetics of in vitro ATC release, we noticed that all articaine-in-NLC curves showed faster release (~ 100% in 8 h) than lidocaine in NLC (14 h^29,37^), confirming the hydrophilic character of ATC. In addition, the initial burst phase caused by non-encapsulated articaine (about 30%), is desirable in the clinical practice to ensure the rapid onset of anesthesia.

The stability of these formulations during 1 year of storage at room temperature, was followed, and no statistically significant variation was noticed in comparison to the initial values of size, PDI and zeta potential (Supplementary Table S9; Supplementary Fig. S13). Once more the obtained results confirmed the literature, showing that NLC are able to encapsulate local anesthetics with good shelf stability^6^.

While factorial design and in vitro release tests gave essential information for the development of NLC, the effectiveness of the optimized formulations had to be tested in order to provide novel parameters for the selection of the best formulation. Since for nanostructured carriers, in vitro experiments could yield results uncorrelated with in vivo ones^41^, the search for alternative in vivo tests are essential, zebrafish being a promising model^15^. Thus, the optimized formulations have been submitted to several tests in zebrafish, to analyze the success of functionalizing the nanoparticles with active lipids, and the benefits of articaine encapsulation.

### In vivo tests in zebrafish larvae

At first, we determined the working concentration of NLC formulations to be the higher concentration not to induce death or phenotypic effects in the body of 5 days post fertilization (dpf) larvae. Since after 1 h post incubation (hpi), only NLC-CO1 and NLC-CO2 formulations caused mortality, at a concentration of 10^13^ nanoparticles/mL, the safety working concentration of 10^12^ nanoparticles/mL was adopted for all formulations. Even when NLCs were loaded with ATC at concentrations up to 1 mM (*ca.* 10^12^ nanoparticles/mL) no larvae death or alterations were registered after 1 hpi, as shown in Supplementary Figures S10A and S11. Just for the 24 hpi (absorption) test, the working concentration was reduced to 10^10^ nanoparticles/mL due to the mortality caused by NLC-CO1 and NLC-CO2 (Supplementary Fig. S10B).

#### NLC absorption by zebrafish larvae

**Figure 2:**
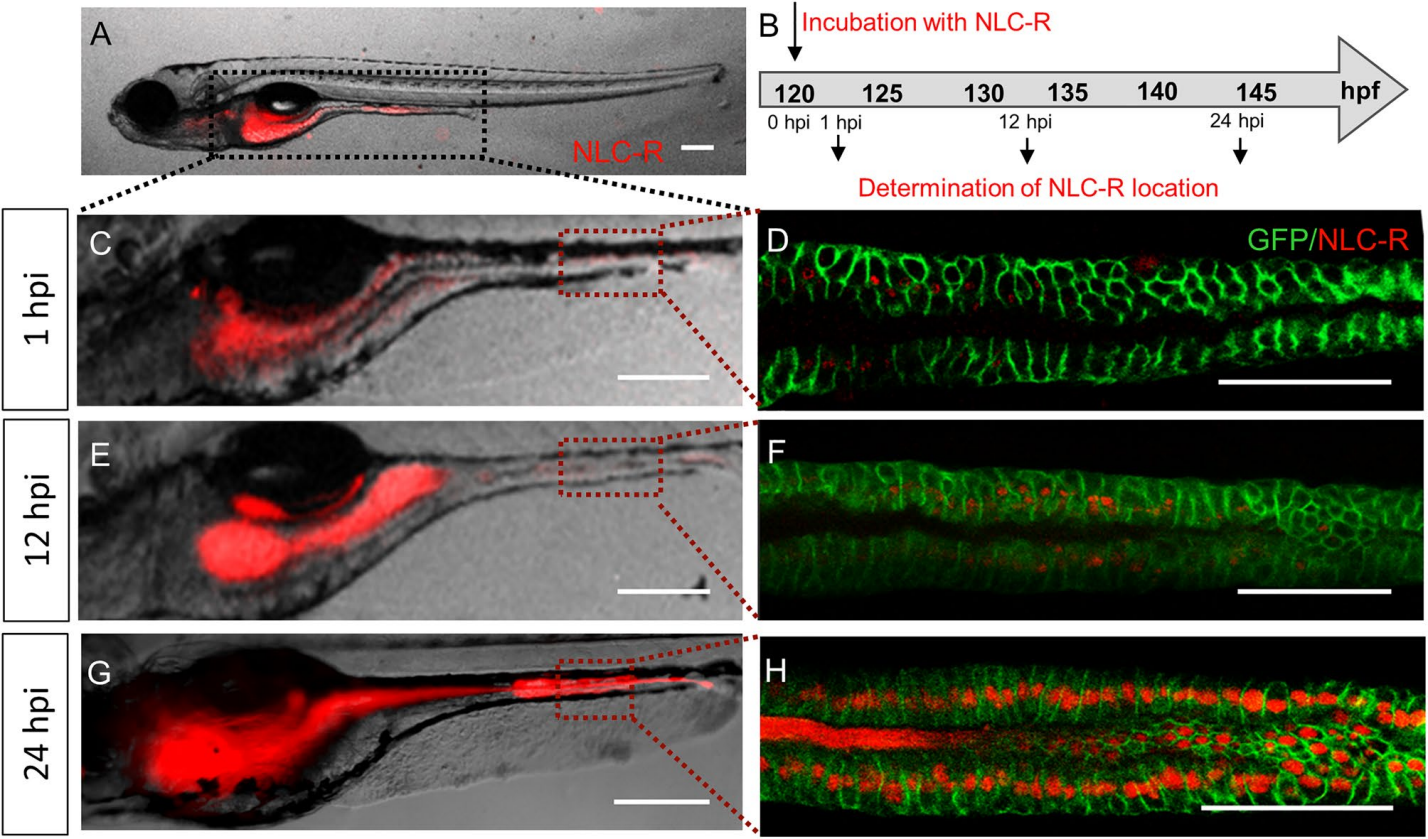
Absorption of NLCs in the larval body. (**A**) *TgBAC(cldn15la:GFP)*^*pd1034*^ larvae, at 5 days post fertilization (dpf), exposed to NLCs loaded with rhodamine-PE, by the immersion method. The larvae intestine is demarked with a black dotted line. (**B**) Timeline: fluorescence was monitored at 1, 12 and 24-h post incubation (hpi). Representative images of larvae examined under a fluorescence stereomicroscope at 1 hpi (**C**), 12 hpi (**E**) and 24 hpi (**G**). A region of the mid-intestine of *TgBAC(cldn15la:GFP)*^*pd1034*^ larvae, demarked by a red dotted line in (**C**,**E**,**G**) was examined under confocal microscope at 1, 12 and 24 hpi (**D**,**F**,**H**, respectively). NLC-R: NLCs labeled with red fluorescent rhodamine-PE. Scale bar 200 μm. The images in **A**, **C**–**H** were edited with ImageJ software v.1.52a (https://imagej.nih.gov/ij/).

To determine if NLCs were absorbed by the larval body, the accumulation of the fluorescent probe (0.01% red fluorescent rhodamine-PE) incorporated (%EE > 99%, Table S7) in NLCs was examined in specific organs of 5 dpf WT larvae at 1-, 12- and 24-hpi (Fig. 2A). As observed in Fig. 2, in all analyzed times the fluorescent NLCs were mainly observed in the gastrointestinal tract. The red fluorescent signal was more intense at 24 hpi, indicating that accumulation was time-dependent (Fig. 2C,E,G). To confirm that NLCs were only present in the intestinal lumen or have been absorbed by the mucosal epithelium, we took advantage of the *TgBAC(cldn15la:GFP)*^*pd1034*^ transgenic line, in which intestinal epithelial cell plasma membrane is fluorescently green-labeled. Due to the detection of fluorescence in the whole intestine, a region was randomly selected to be analyzed. The results in Fig. 2D,F,H suggest that NLCs (red) were indeed internalized by epithelial cells (green), in a time dependent manner. Moreover, no differences were seen between formulations with and without ATC. These results are in agreement with previous reports indicating that the intestinal epithelium is an important route for particle uptake by larvae^42–44^. However, in a few larvae, fluorescence was also observed on the posterior-ventral area of head, probably in the esophagus (Supplementary Fig. S12A–C).

#### Anti-inflammatory activity of NLC excipients

**Figure 3:**
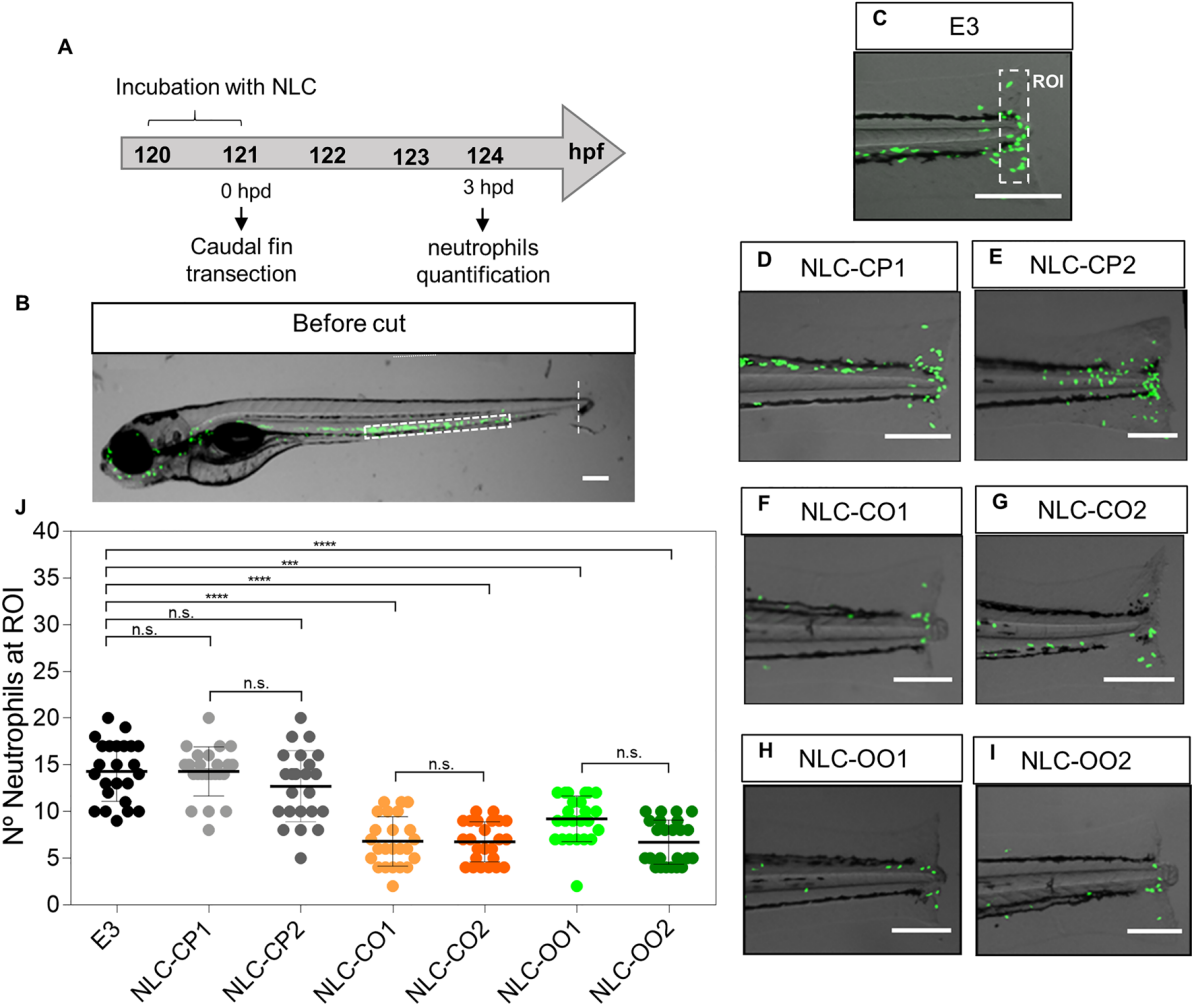
NLC excipients anti-inflammatory activity in zebrafish larvae. **(A**,**B**) Experimental strategy and representative image of a *Tg(BACmpx:GFP)*^*i114*^ larvae before damage. (**C**–**I**) Representative images of the damaged area of nontraded larvae (**C**) or treated (**D**–**I**) with different NLCs. (**J**) Quantification of the number of neutrophils present at the damaged zone (Region of interest (ROI)) at 3 h post damage (hpd) from 3 independent experiments (n = 24). Scale bar 200 μm. Statistical analysis: one-way ANOVA and Tukey post-hoc test. Statistical significance: ***p < 0.001, ****p < 0.0001; *n.s.* not significant. The images (**B**) and (**C**–**I**) were edited using ImageJ software v.1.52a (https://imagej.nih.gov/ij/).

To evaluate the anti-inflammatory properties of natural excipients, we used a well stablished acute inflammation model in zebrafish, which involves caudal fin transection^33,45,46^. For that, 5dpf *Tg(BACmpx:GFP)*^*i114*^ larvae were incubated in suspensions with the different NLC formulations for 1 h and then caudal fin transection was performed. Neutrophils recruitment to the affected area was analyzed at 3 h post damage (hpd) (Fig. 3A), when the highest recruitment was observed (data not shown). Since, during homeostasis, neutrophils are localized mainly in the caudal hematopoietic tissue (white rectangle in Fig. 3B) their migration to the damaged zone can be used as an inflammation marker. The results obtained for the control group showed an average of 15 neutrophils infiltrated in the damaged zone^33^ (Fig. 3C,J), with no significant differences to those larvae treated with NLC-CP1 and NLC-CP2—average of 15 and 13 neutrophils, respectively, at the affected area (Fig. 3D,E,J). On the contrary, in larvae treated with NLC-CO1, NLC-CO2, NLC-OO2 and, NLC-OO1, the number of neutrophils at the injury was significantly lower: an average of 6 (NLC-CO1, NLC-CO2 and NLC-OO2) and 8 neutrophils (NLC-OO1), as shown in Fig. 3F–J.

These results demonstrate that NLCs prepared with copaiba or olive oil have a significant anti-inflammatory effect, in accordance with the literature. Indeed, previous experiments in rodents using a motor cortex injury as inflammatory model showed a decrease in neutrophil migration when the animals were treated with copaiba oil^47^. In the case of olive oil, leukocyte adhesion has been profoundly inhibited during an LPS-induced inflammation test, in mice^48^.

Although the above test confirmed the successful functionalization of nanoparticles, it was not useful to reveal the best formulation, since all functional-NLCs (NLC-CO1, NLC-CO2, NLC-OO1 and NLC-OO2) displayed an anti-inflammatory effect. In addition, those formulations that differed in composition only by surfactants [NLC-CP1 (P68) vs. NLC-CP2 (T80), and NLC-OO1 (P68) vs. NLC-OO2 (T80)] showed equivalent anti-inflammatory activity.

#### In vivo screening of the effect of NLC-loaded with articaine

The screening of biological (antinociception) effects elicited by local anesthetics in lipid-based drug delivery formulations—e.g. paw-pressure, pinpricking, hot-plate, infraorbital nerve block tests^49^—is mostly carried out in rodents. As recently reviewed, these tests have their own drawbacks as pre-clinical anesthesia studies^6^ and, unlike the zebrafish model, all of them allow a restricted number of formulations to be simultaneously analyzed. Another advantage of zebrafish model, when used in the study of local anesthetics, is the easy access to the cardiac system function, where LA promote bradycardia by blocking cardiomyocytes voltage-dependent channels^50^. So, the bradycardia effect in zebrafish has been used to evaluate the anesthetic activity of different compounds such as lidocaine, propofol and isoflurane^51^. Indeed, despite ATC being a local anesthetic, the observed effect in zebrafish is systemic, since the LA is administered in the larvae’s bathing solution^52^. Also, the systemic toxicity of local anesthetics is well documented^1^. By the same reasoning, LA effect on excitable membranes in the central nervous system (CNS) could be evaluated through the touch-response test ^53^.

##### Bradycardia and anesthesia

**Figure 4:**
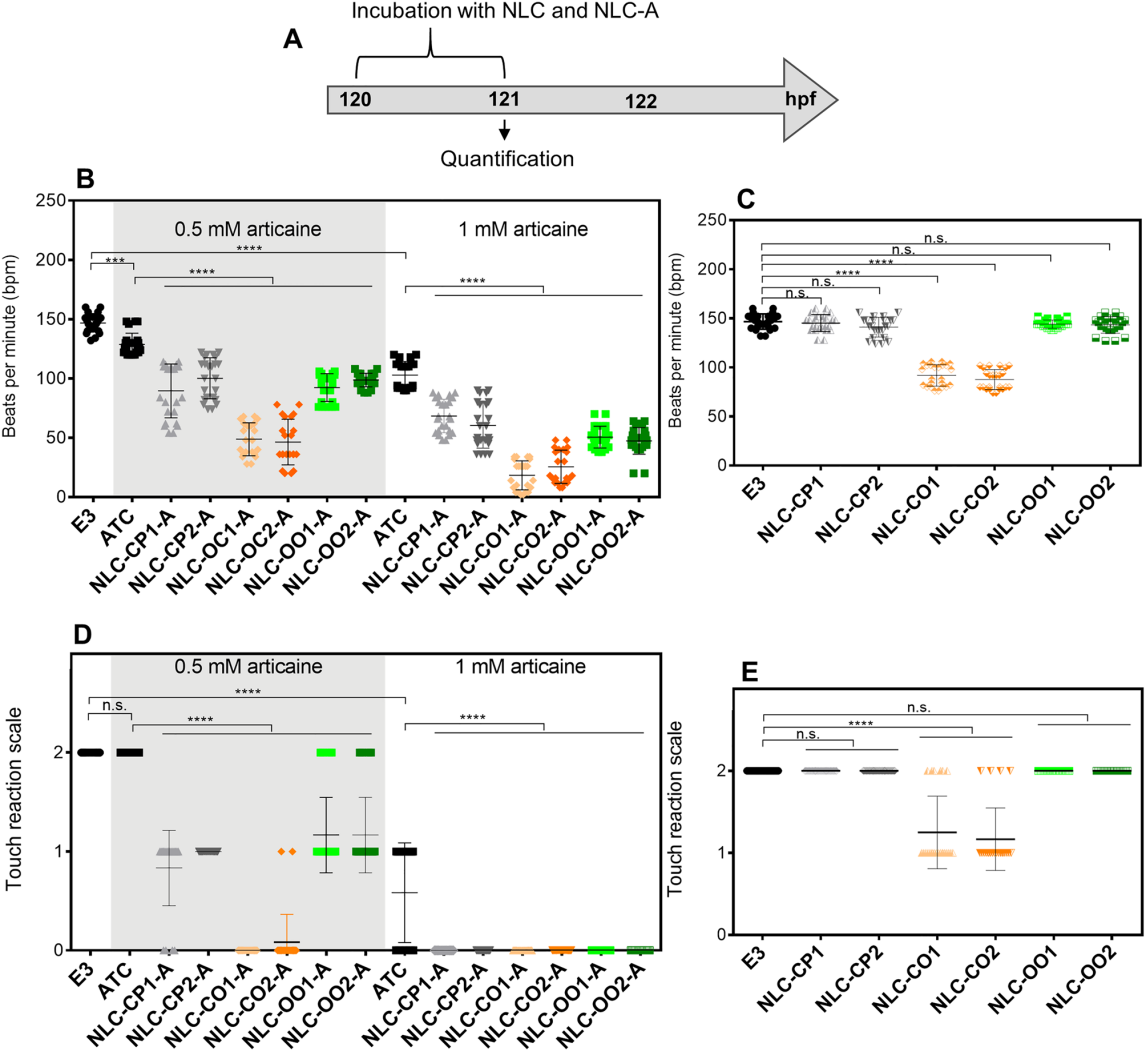
(**A**) Experimental strategy. (**B**) Bradycardia after 1 h of incubation of free articaine (ATC) and articaine-loaded nanostructured lipid carriers at concentrations of 0.5 and 1 mM and (**C**) their respective controls without articaine. (**D**) Touch reaction after 1 h incubation of ATC and articaine-loaded nanostructured lipid carriers at concentrations of 0.5 and 1 mM and (**E**) their respective controls without articaine. Touch reaction scale: Score 0 means larva did not react, score 1 means larva react between 1 and 3 s after touch, and score 2 means larva react in less than 1 s after touch. n = 24. E3 = medium only, no treatment (control). Statistical analysis: one-way ANOVA and Tukey post-hoc test. Statistical significance: ***p < 0.001, ****p < 0.0001; *n.s.* non-significant.

As shown in Fig. 4B, 5 dpf larvae incubated in E3 showed an average of 150 beats per minute (bpm), consistent with previous reports^54^. After 1 h post incubation, the two concentrations (0.5 and 1.0 mM) of free ATC significantly decreased the heart rate compared to control larvae to 130 and 100 bpm, respectively. The synthetic NLCs (NLC-CP1-A and NLC-CP2-A) and those produced with olive oil (NLC-OO1-A and NLC-OO2-A) intensified (> 30%) the bradycardia effect in relation to free ATC in the two tested concentration, suggesting that encapsulation potentiates the anesthetic effect of ATC, as already observed with lidocaine^37^. The bradycardia effect was even more evident (> 60% in comparison to free ATC) with copaiba-based NLCs (NLC-CO1-A and NLC-CO2-A) (Fig. 4B). The control NLCs (without articaine) did not affect the heart rate, except for the those composed of copaiba oil, that decreased heartbeats to 92 and 88 bpm, respectively (NLC-CO1, NLC-CO2; Fig. 4C). Control NLCs were also tested simultaneously to free ATC and again only the nanoparticles with copaiba oil showed synergistic effects (bradycardia) to the anesthetic.

In relation to the touch response, free ATC decreased the larvae escape response only at the highest (1 mM) concentration tested (Fig. 4D), in agreement with previous reports for lidocaine^55^. At 0.5 mM articaine, the NLCs prepared with synthetic lipids (NLC-CP1-A and NLC-CP2-A) and those composed of olive oil (NLC-OO1-A and NLC-OO2-A) potentiated by 50% the anesthetic effect in comparison to free ATC. Moreover, at the same concentration, NLCs composed of copaiba oil (NLC-CO1-A, NLC-CO2-A) exhibited the most pronounced effect over the CNS, inducing 100% anesthesia of the larvae. As observed for the bradycardia effect, control NLCs formulations prepared with copaiba oil (NLC-CO1, NLC-CO2; Fig. 4E) exhibited some anesthetic effect, confirming that copaiba oil played a synergistic effect with ATC.

It is common in the medical practice to use the local anesthetic lidocaine (either intravenously or intramuscularly and at precise concentrations) as an antiarrhythmic agent^56^ because of its vasodilator activity and depression of myocardial contraction^50^. In many aspects, zebrafish heart physiology is similar to that of humans^57^, therefore, free ATC (like lidocaine^51^) caused a depression in heartbeats of zebrafish larvae (bradycardia). Moreover, when encapsulated in NLC, ATC promoted deeper bradycardia in relation to its free form, suggesting an improved delivery and potentiation of the anesthetic activity, as already reported for lidocaine^40^.

The copaiba oil present in control NLCs was also able to induce bradycardia and adjuvant anesthesia in the CNS. This result agrees with the literature that reports that copaiba oil binds to opioid receptors^58^, explaining the depressant effects over the myocardium and nervous system. Similar potentiation of the anesthetic effect has been observed when other drugs were co-encapsulated with local anesthetic (e.g. tetrodotoxin, saxitoxins and dexamethasone)^59^. It is worth noting that, regardless of the surfactant used to prepare the copaiba-oil NLCs, their biological response did not change.

##### Bradycardia recovery

**Figure 5:**
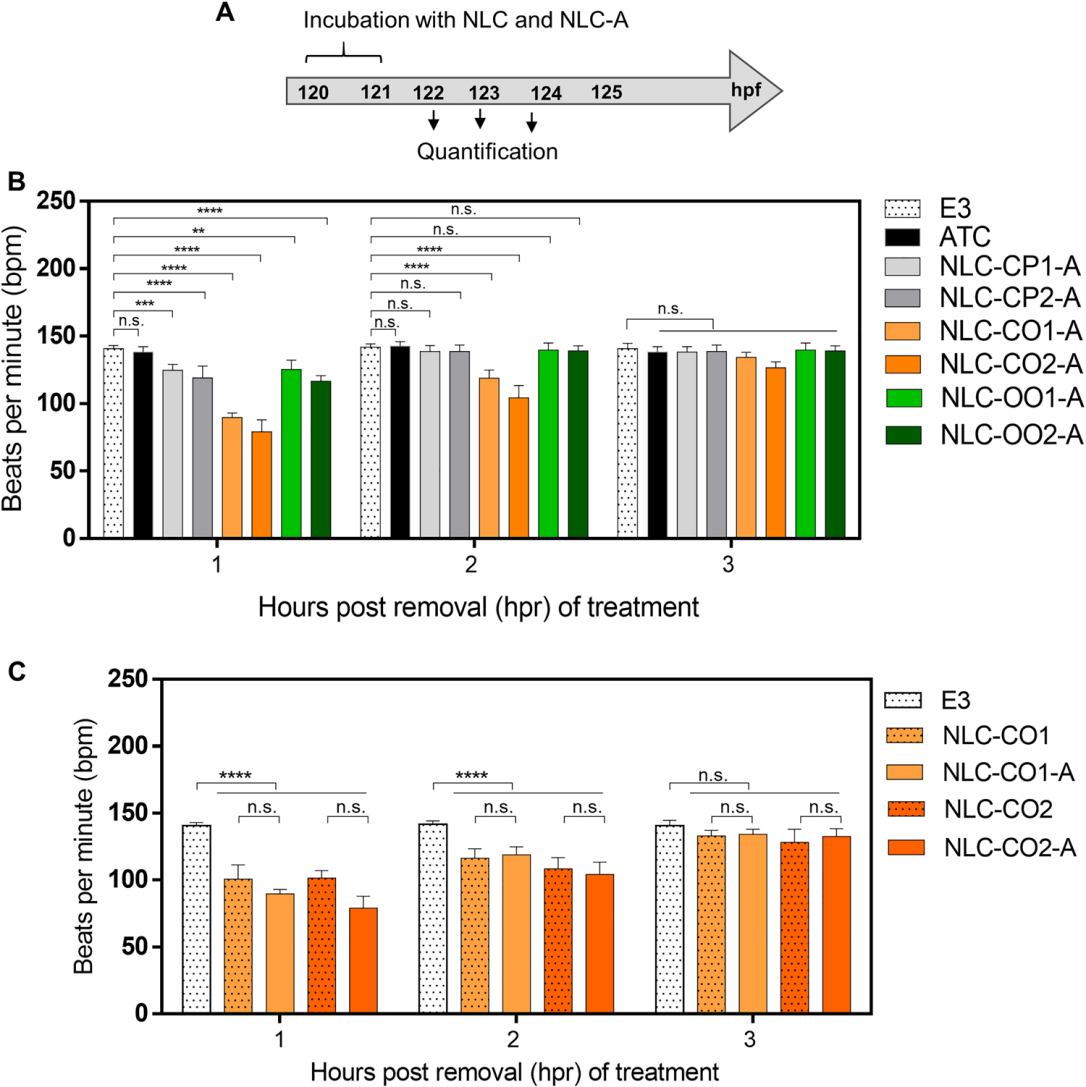
Bradycardia recovery. **(A**) Experimental strategy. (**B**) Heartbeats counted after 1, 2 and 3 h post removal of treatment with 1 mM articaine, free or in NLC formulations. (**C**) Comparison of the effect of copaiba oil-NLC formulations, with and without articaine on the heartbeat. n = 24. Statistical analysis performed by one-way ANOVA and Tukey post-hoc test. Statistical significance: **p < 0.01, ***p < 0.001, ****p < 0.0001; *n.s.* non-significant.

Since in vitro tests evidenced prolonged drug release from the NLCs, we measured the bradycardia-recovery time to check if encapsulation of ATC would affect such depressor effect. For that, 5 dpf larvae were exposed to 1 mM ATC (dose for the highest bradycardia effect observed in Fig. 4) either free or encapsulated into the NLC formulations. After 1 h the medium was removed and returned to normal (E3) medium and the heart rate was analyzed at 1-, 2- or 3-h post ATC removal (hpr) (Fig. 5A). At 1 hpr the larvae incubated with free ATC returned to the basal heartbeats rate (Fig. 5B), while larvae treated with ATC encapsulated in NLCs were still bradycardic. At 2 hpr only NLC-CO1 and NLC-CO2 still maintained a significantly reduced heart rate: 120 bpm and 100 bpm, respectively. At 3 hpr all treatments have lost the bradycardia effect. These in vivo results, in accordance with the in vitro release experiment, confirmed that encapsulation in NLCs prolonged the effect of ATC.

In the same test, Fig. 5C emphasizes the results obtained with NLCs composed of copaiba oil, with and without ATC. In this case, unlike observed with the other NLC formulations, the bradycardia effect surpassed 2 hpr, possibly due to copaiba oil effect on opioid receptors^58^ as evinced from the prolonged bradycardia observed with control NLCs, without ATC (NLC-CO1 and NLC-CO2). Moreover, after 1 hpr, the bradycardia effects of NLC-CO1-A and NLC-CO2-A were only 2 and 7% higher than NLCs without ATC (NLC-CO1 and NLC-CO2, respectively). Therefore, the bradycardia effect of copaiba oil seemed to prevail upon that of ATC. Once more no significant differences were found between NLCs that differ in their composition only by the surfactant: NLC-CP1 (P68) vs. NLC-CP2 (T80), and NLC-OO1 (P68) vs. NLC-OO2 (T80) (Fig. 5B). Although the literature indicates that Pluronic surfactants increases NLC circulation time in vivo by sterically reducing the action of blood lipases over their lipid constituents^60^, in this work we saw no differences between the two surfactants tested (Tween 80 and Pluronic F68) in any of the measured zebrafish in vivo responses.

### Formulation selection

In the development of the NLCs we have performed FD optimization, characterization of the formulations and, through an innovative process, several tests in zebrafish in order to select the best composition for the desired application. Table 5 summarizes the information collected for each of the studied formulations.

**Table 5:** Physicochemical properties and biological responses of the NLC formulations developed for articaine.

	Physicochemical properties				Biological responses			
Formulation	Size (nm)	PDI	ZP (mV)	%EE	Anti-inflammatory property	Bradycardia (bpm ± SD)^a^	Bradycardia-recovery time (h)^b^	Anesthesia improvement (% ± SD) ^c^
NLC-CP1-A	237.0 ± 0.4	0.167 ± 0.001	− 38.3 ± 0.5	74.8 ± 1.2	No	68 ± 4	2	58 ± 6
NLC-CP2-A	237.6 ± 3.3	0.169 ± 0.015	− 42.1 ± 0.5	66.8 ± 2.3	No	60 ± 6	2	50 ± 0
NLC-CO1-A	256.4 ± 0.2	0.200 ± 0.009	− 43.7 ± 0.6	70.5 ± 1.4	Yes	18 ± 3	3	100 ± 0
NLC-CO2-A	217.7 ± 0.8	0.174 ± 0.004	− 40.2 ± 1.1	70.6 ± 1.8	Yes	25 ± 4	3	100 ± 4
NLC-OO1-A	254.1 ± 1.8	0.190 ± 0.005	− 42.2 ± 0.5	68.3 ± 1.9	Yes	50 ± 3	2	54 ± 6
NLC-OO1-A	243.3 ± 5.8	± 0.176 ± 0.022	− 49.7 ± 0.7	68.0 ± 2.5	Yes	47 ± 3	2	54 ± 6

Size = nanoparticle diameter, in nm; *PDI* polydispersity index, ZP zeta potential, in mV, %EE percent encapsulation efficiency of articaine, bpm heart beats per minute.

^a^With 1 mM ATC, after 1 hpi; ^b^with 1 mM ATC; ^c^formulations containing 0.5 mM ATC, in relation to free ATC at the same concentration.

Among the formulations, those with the best biological responses were the NLCs composed by copaiba oil, because of their anti-inflammatory activity and increased anesthetic effect (potency and time of action). These formulations (NLC-CO1-A and NLC-CO2-A) differ from each other only by the surfactant: P68 or T80, respectively. No statistical differences were detected in the biological responses elicited by the formulations prepared with these two surfactants. So, to select one, we considered the physicochemical properties of formulations and surfactants. The formulation with T80 exhibited smaller sizes and PDI, that literature points out as better parameters for parenteral administration, which is our goal. In addition, T80 is cheaper than P68, favoring its future scale up production and commercialization. In this sense, the formulation composed of cetyl palmitate with copaiba oil and Tween 80 (NLC-CO2-A) was selected as the best one for future tests in mammal models of inflammatory pain.

Therefore, the coupling of factorial design with zebrafish model allowed us—still in the NLC development stage—to determine in a simple and fast way the biological responses of optimized formulations, avoiding future waste of time and money. In addition, such results would never be achieved with in vitro (e.g. cell cultures) approaches that are not able to mimic the complexity of whole organisms^41^.

## Conclusion

Among several excipient options for developing an articaine-in-NLC formulation, we selected that composed of cetyl palmitate plus copaiba oil and Tween 80, since it showed the smallest size and PDI (better for parenteral use), anti-inflammatory activity of excipients and potentiated anesthetic effect. This NLC formulation has a great potential for clinical application in dentistry, for the pain management in tissues where an inflammatory process is installed. Besides, it has also the potential to become an effective protocol in dairy dentistry practice, or in the control of surgical/postsurgical pain (due to the prolonged release). In addition, since NLC are effective for topical application, we foresee other therapeutic uses for this formulation, such as in anesthesia of the skin.

In addition, we showed that tests conducted with zebrafish can be coupled with factorial design in the optimization of lipid-based drug delivery systems, reducing time and costs of pharmaceutical development. Also, our results open possibilities for several other studies on the interaction of nanoparticles with biological systems, to which the zebrafish model can bring advances.

## Supplementary information

Supplementary Information.
