# Supplementary material for: Articaine in functional NLC show improved anesthesia and anti-inflammatory activity in zebrafish
Source: Sci Rep. 2020 Nov 12;10:19733. doi: 10.1038/s41598-020-76751-6 (PMC7665027; doi:10.1038/s41598-020-76751-6)
Supplement: Supplementary file 1 — Supplementary Information. [file 41598_2020_76751_MOESM1_ESM.docx]

**Articaine in functional NLC show improved anesthesia and anti-inflammatory activity in zebrafish**

Gustavo H. Rodrigues da Silva, Gabriela Geronimo, Juan P. García-López, Lígia N. M. Ribeiro, Ludmilla D. de Moura, Márcia C. Breitkreitz, Carmen G. Feijoó, Eneida de Paula

**SUPPLEMENTARY INFORMATION**

**Table S1**. Composition of the formulations and responses obtained in the 2^3^ experimental design for encapsulation of articaine in NLC using cetyl palmitate, Dhaykol 6040 and Pluronic F-68 as excipients.

|  | **Variables** | | | **Responses** | | |
| --- | --- | --- | --- | --- | --- | --- |
| **Sample** | **Total lipids**  **(% w/w)** | **Pluronic F-68**  **(% w/w)** | **ATC**  **(% w/w)** | **Size**  **(nm)** | **PDI** | **Zeta potential**  **(mV)** |
| 6 | 9 | 2.5 | 0 | 293.2 | 0.222 | -33.3 |
| 3 | 15 | 2.5 | 0 | 263.6 | 0.195 | -35.1 |
| 1 | 9 | 5 | 0 | 271.7 | 0.174 | -31.9 |
| 7 | 15 | 5 | 0 | 231.3 | 0.188 | -36.8 |
| 11 | 9 | 2.5 | 4 | 271.5 | 0.184 | -44.1 |
| 2 | 15 | 2.5 | 4 | 303.2 | 0.176 | -46.3 |
| 9 | 9 | 5 | 4 | 182.7 | 0.144 | -40.9 |
| 4 | 15 | 5 | 4 | 218.4 | 0.160 | -41.8 |
| 10 | 12 | 3.75 | 2 | 226.0 | 0.181 | -45.3 |
| 5 | 12 | 3.75 | 2 | 225.0 | 0.160 | -40.9 |
| 8 | 12 | 3.75 | 2 | 230.0 | 0.169 | -45.8 |

**Table S2**. Composition of the formulations and responses obtained in the 2^3^ experimental design for encapsulation of articaine in NLC using cetyl palmitate, Dhaykol 6040 and Tween 80 as excipients.

|  | **Variables** | | | **Responses** | | |
| --- | --- | --- | --- | --- | --- | --- |
| **Sample** | **Total lipids**  **(% w/w)** | **Tween 80**  **(% w/w)** | **ATC**  **(% w/w)** | **Size**  **(nm)** | **PDI** | **Zeta potential**  **mV** |
| 8 | 9 | 2.5 | 0 | 405.7 | 0.205 | -33.4 |
| 3 | 15 | 2.5 | 0 | 585.0 | 0.036 | -33.8 |
| 9 | 9 | 5 | 0 | 309.4 | 0.230 | -33.3 |
| 11 | 15 | 5 | 0 | 281.2 | 0.176 | -32.1 |
| 7 | 9 | 2.5 | 4 | 326.9 | 0.095 | -43.7 |
| 5 | 15 | 2.5 | 4 | 480.2 | 0.208 | -42.3 |
| 2 | 9 | 5 | 4 | 293.3 | 0.163 | -47.0 |
| 4 | 15 | 5 | 4 | 297.2 | 0.138 | -42.4 |
| 1 | 12 | 3.75 | 2 | 296.0 | 0.157 | -43.8 |
| 10 | 12 | 3.75 | 2 | 282.6 | 0.157 | -44.4 |
| 6 | 12 | 3.75 | 2 | 237.6 | 0.169 | -42.1 |

**Table S3**. Composition of the formulations and responses obtained in the 2^3^ experimental design for encapsulation of articaine in NLC using avocado butter, copaiba oil and Pluronic F-68 as excipients.

|  | **Variables** | | | **Responses** | | |
| --- | --- | --- | --- | --- | --- | --- |
| **Sample** | **Total lipids**  **(% w/w)** | **Pluronic F-68**  **(% w/w)** | **ATC**  **(% w/w)** | **Size**  **(nm)** | **PDI** | **Zeta potential**  **mV** |
| 8 | 9 | 2.5 | 0 | 308.1 | 0.329 | -31.7 |
| 3 | 15 | 2.5 | 0 | 364.8 | 0.281 | -31.8 |
| 9 | 9 | 5 | 0 | 223.7 | 0.204 | -28.4 |
| 11 | 15 | 5 | 0 | 237.1 | 0.179 | -29.7 |
| 7 | 9 | 2.5 | 4 | 271.1 | 0.194 | -47.8 |
| 5 | 15 | 2.5 | 4 | 307.5 | 0.198 | -49.1 |
| 2 | 9 | 5 | 4 | 231.2 | 0.181 | -48.0 |
| 4 | 15 | 5 | 4 | 238.8 | 0.170 | -46.8 |
| 1 | 12 | 3.75 | 2 | 238.2 | 0.170 | -46.8 |
| 10 | 12 | 3.75 | 2 | 250.6 | 0.197 | -46.6 |
| 6 | 12 | 3.75 | 2 | 240.8 | 0.178 | -45.0 |

**Table S4**. Composition of the formulations, and responses obtained in the 2^3^ experimental design for encapsulation articaine in NLC composed of avocado butter, copaiba oil and Tween 80 as excipients.

|  | **Factors** | | | **Responses** | | |
| --- | --- | --- | --- | --- | --- | --- |
| **Sample** | **Total lipids**  **(% w/w)** | **Tween 80**  **(% w/w)** | **ATC**  **(% w/w)** | **Size**  **(nm)** | **PDI** | **Zeta potential**  **mV** |
| 8 | 9 | 2.5 | 0 | 236.4 | 0.181 | -31.3 |
| 3 | 15 | 2.5 | 0 | 282.0 | 0.210 | -29.3 |
| 9 | 9 | 5 | 0 | 185.8 | 0.164 | -27.5 |
| 11 | 15 | 5 | 0 | 266.3 | 0.181 | -30.5 |
| 7 | 9 | 2.5 | 4 | 203.9 | 0.165 | -38.3 |
| 5 | 15 | 2.5 | 4 | 268.1 | 0.158 | -41.0 |
| 2 | 9 | 5 | 4 | 187.6 | 0.147 | -36.4 |
| 4 | 15 | 5 | 4 | 211.6 | 0.164 | -40.8 |
| 1 | 12 | 3.75 | 2 | 219.3 | 0.151 | -40.2 |
| 10 | 12 | 3.75 | 2 | 207.7 | 0.153 | -38.5 |
| 6 | 12 | 3.75 | 2 | 217.7 | 0.174 | -39.0 |

**Table S5**. Composition of the formulations and responses obtained in the 2^3^ experimental design for encapsulation of articaine in NLC using avocado butter, olive oil and Pluronic F-68 as excipients.

|  | **Factors** | | | **Responses** | | |
| --- | --- | --- | --- | --- | --- | --- |
| **Sample** | **Total lipids**  **(% w/w)** | **Pluronic F-68**  **(% w/w)** | **ATC**  **(% w/w)** | **Size**  **(nm)** | **PDI** | **Zeta potential**  **mV** |
| 8 | 9 | 2.5 | 0 | 254.3 | 0.169 | -32.1 |
| 3 | 15 | 2.5 | 0 | 227.8 | 0.187 | -33.8 |
| 9 | 9 | 5 | 0 | 283.4 | 0.160 | -36.4 |
| 11 | 15 | 5 | 0 | 216.8 | 0.173 | -29.3 |
| 7 | 9 | 2.5 | 4 | 278.8 | 0.231 | -46.5 |
| 5 | 15 | 2.5 | 4 | 322.6 | 0.194 | -47.8 |
| 2 | 9 | 5 | 4 | 229.8 | 0.171 | -46.2 |
| 4 | 15 | 5 | 4 | 262.5 | 0.179 | -44.6 |
| 1 | 12 | 3.75 | 2 | 254.1 | 0.190 | -42.2 |
| 10 | 12 | 3.75 | 2 | 250.3 | 0.184 | -45.5 |
| 6 | 12 | 3.75 | 2 | 254.4 | 0.179 | -45.3 |

**Table S6**. Composition of the formulations and responses obtained in the 2^3^ experimental design for encapsulation of articaine in NLC using avocado butter, olive oil and Tween 80 as excipients.

|  | **Factors** | | | **Responses** | | |
| --- | --- | --- | --- | --- | --- | --- |
| **Sample** | **Total lipids**  **(% w/w)** | **Tween 80**  **(% w/w)** | **ATC**  **(% w/w)** | **Size**  **(nm)** | **PDI** | **Zeta potential**  **mV** |
| 8 | 9 | 2.5 | 0 | 252.2 | 0.196 | -33.2 |
| 3 | 15 | 2.5 | 0 | 302.1 | 0.244 | -33.8 |
| 9 | 9 | 5 | 0 | 204.6 | 0.201 | -32.4 |
| 11 | 15 | 5 | 0 | 250.7 | 0.189 | -32.5 |
| 7 | 9 | 2.5 | 4 | 264.5 | 0.148 | -41.4 |
| 5 | 15 | 2.5 | 4 | 282.6 | 0.184 | -51.9 |
| 2 | 9 | 5 | 4 | 260.9 | 0.080 | -46.8 |
| 4 | 15 | 5 | 4 | 243.9 | 0.133 | -48.9 |
| 1 | 12 | 3.75 | 2 | 250.4 | 0.234 | -46.1 |
| 10 | 12 | 3.75 | 2 | 243.3 | 0.176 | -49.7 |
| 6 | 12 | 3.75 | 2 | 265.6 | 0.191 | -47.3 |

**Table S7**. Characterization of NLCs prepared with rhodamine-PE by Dynamic Light Scattering (size, PDI, zeta potential) and Nanotracking analysis (nanoparticles concentration) plus articaine encapsulation efficiency.

| **Formulation** | **Size**  **(nm)** | **PDI** | **Zeta**  **potential (mV)** | **Nanoparticles concentration**  **(.10^13^ / mL)** | **%EE**  **Rhodamine-PE** | |
| --- | --- | --- | --- | --- | --- | --- |
| NLC-CP1 + rhodamine PE | 233.6 ± 0.1 | 0.167 ± 0.015 | -31.6 ± 0.5 | 6.8 ± 0.9 | 99.1 ± 0.5 | |
| NLC-CP1-A + rhodamine PE | 279.8 ±2.1 | 0.145 ±0.048 | -39.9 ±0.7 | 7.2 ± 0.5 | 99.2 ± 0.3 | |
| NLC-CP2 + rhodamine PE | 227.9 ± 1.6 | 0.191 ± 0.039 | -27.0 ± 0.4 | 8.7 ± 1.0 | 99.3 ± 0.6 | |
| NLC-CP2-A + rhodamine PE | 221.8 ±2.9 | 0.177 ±0.003 | -41.3 ±1.4 | 9.1 ± 0.4 | 99.3 ± 0.4 | |
| NLC-CO1 + rhodamine PE | 227.6 ± 0.3 | 0.143 ± 0.041 | -30.7 ± 0.8 | 7.6 ± 0.1 | 99.5 ± 0.3 | |
| NLC-CO1-A + rhodamine PE | 251.4 ±1.8 | 0.182 ±0.019 | -45.8 ±0.2 | 7.7 ± 0.8 | 99.3 ± 0.2 | |
| NLC-CO2 + rhodamine PE | 203.9 ± 1.6 | 0.175 ± 0.020 | -21.3 ± 0.6 | 9.2 ± 0.2 | 99.2 ± 0.5 | |
| NLC-CO2-A + rhodamine PE | 201.8 ±4.7 | 0.149 ±0.027 | -34.5 ±0.7 | 9.5 ± 0.9 | 99.7 ± 0.4 | |
| NLC-OO1 + rhodamine PE | 236.4 ± 1.9 | 0.167 ± 0.048 | -31.4 ± 0.6 | 6.3 ± 0.5 | 99.5 ± 0.4 | |
| NLC-OO1-A + rhodamine PE | 271.5 ±1.9 | 0.175 ±0.028 | -48.5 ±0.8 | 6.9 ± 0.7 | 99.6 ± 0.5 | |
| NLC-CO2 + rhodamine PE | 229.8 ± 1.7 | 0.207 ± 0.008 | -23.9 ± 0.8 | 5.4 ± 0.6 | 99.0 ± 0.3 | |
| NLC-CO2-A + rhodamine PE | 233.1 ±0.6 | 0.139 ±0.002 | -45.0 ±0.1 | 6.0 ± 0.2 | 99.5 ± 0.6 |  |

**Table S8**. R^2^ (coefficient of determination) of different mathematical models applied to the release kinetic curves, as calculated by the KinetDS software ^27^. See Table 1 for abbreviation and NLC composition.

|  | **Coefficient of determination** | | | | | | |
| --- | --- | --- | --- | --- | --- | --- | --- |
| **Models:** | **Zero**  **order** | | **First**  **order** | **Korsmeyer-Peppas** | **Weibull** | **Hixson-Crowell** | **Higuchi** |
|  |  |  |  |  |  |  |  |
| **Samples:** |  |  |  |  |  |  |  |
| NLC-CP1-A | 0.4798 | 0.0842 | | 0.9896 | 0.9975 | 0.2811 | -1.1989 |
| NLC-CP2-A | 0.3909 | 0.0806 | | 0.9878 | 0.9967 | 0.2326 | -1.4958 |
| NLC-CO1-A | 0.3910 | 0.0784 | | 0.9867 | 0.9969 | 0.2285 | -1.5025 |
| NLC-CO2-A | 0.3588 | 0.0769 | | 0.9859 | 0.9983 | 0.2168 | -1.5389 |
| NLC-OO1-A | 0.4059 | 0.0799 | | 0.9871 | 0.9980 | 0.2423 | -1.3726 |
| NLC-OO2-A | 0.4148 | 0.0806 | | 0.9880 | 0.9950 | 0.2497 | -1.3247 |

**Table S9.** Results of stability tests with the optimized formulations and their controls (without articaine) over a year of storage at room temperature: size (nm) and standard deviation (SD). The composition of each of the 6 NLC formulations can be found in Table 1.

|  | **NLC-CP1** | | **NLC-CP1-A** | | **NLC-CP2** | | **NLC-CP2-A** | | **NLC-CO1** | | **NLC-CO1-A** | | **NLC-CO2** | | **NLC-CO2-A** | | **NLC-OO1** | | **NLC-OO1-A** | | **NLC-OO2** | | **NLC-OO2-A** | |
| --- | --- | --- | --- | --- | --- | --- | --- | --- | --- | --- | --- | --- | --- | --- | --- | --- | --- | --- | --- | --- | --- | --- | --- | --- |
| **Month** | **Value** | **SD** | **Value** | **SD** | **Value** | **SD** | **Value** | **SD** | **Value** | **SD** | **Value** | **SD** | **Value** | **SD** | **Value** | **SD** | **Value** | **SD** | **Value** | **SD** | **Value** | **SD** | **Value** | **SD** |
| **Size (nm)** | | | | | | | | | | | | | | | | | | | | | | | | |
| 0 | 221.9 | 2.8 | 236.9 | 0.5 | 240.9 | 3.0 | 241.2 | 4.2 | 231.9 | 2.2 | 252.9 | 4.2 | 216.8 | 1.7 | 208.8 | 1.6 | 231.8 | 2.3 | 247.1 | 0.8 | 246.5 | 2.4 | 245.8 | 1.8 |
| 1 | 229.2 | 1.5 | 236.5 | 2.8 | 239.0 | 6.2 | 236.3 | 1.3 | 215.6 | 4.1 | 260.8 | 1.5 | 220.8 | 1.6 | 216.3 | 4.5 | 250.3 | 0.6 | 246.6 | 1.4 | 250.3 | 1.9 | 243.3 | 2.0 |
| 2 | 224.1 | 2.8 | 235.7 | 2.1 | 240.7 | 3.4 | 230.3 | 1.2 | 220.6 | 1.4 | 256.9 | 2.0 | 217.7 | 5.0 | 213 | 5.8 | 235.5 | 3.4 | 242.9 | 1.5 | 248.2 | 1.9 | 235.7 | 2.9 |
| 3 | 224.2 | 2.9 | 236.8 | 1.3 | 241.1 | 5.1 | 238.3 | 2.2 | 221.0 | 2.1 | 260.9 | 5.1 | 219.8 | 0.9 | 219.4 | 1.8 | 236.7 | 0.3 | 244.8 | 1.0 | 254.2 | 0.2 | 246.0 | 2.0 |
| 4 | 227.8 | 1.2 | 237.5 | 1.3 | 249.2 | 0.7 | 239.9 | 1.2 | 213.5 | 1.3 | 260.0 | 1.9 | 218.6 | 2.1 | 214.0 | 1.2 | 237.3 | 1.2 | 250.5 | 1.0 | 246.5 | 2.0 | 241.7 | 2.7 |
| 5 | 222.4 | 4.2 | 242.7 | 1.7 | 254.2 | 0.0 | 244.6 | 2.8 | 216.5 | 3.5 | 258.4 | 6.2 | 217.8 | 3.8 | 217.1 | 0.1 | 236.1 | 0.6 | 245.5 | 1.1 | 244.3 | 4.0 | 241.6 | 0.7 |
| 6 | 229.0 | 1.1 | 239.3 | 4.6 | 254.1 | 0.5 | 243.6 | 5.7 | 214.5 | 3.5 | 251.9 | 4.0 | 218.9 | 1.4 | 210.1 | 1.6 | 235.7 | 1.7 | 237.8 | 3.4 | 240.5 | 0.3 | 240.5 | 5.7 |
| 7 | 226.8 | 0.4 | 243.3 | 1.3 | 255.3 | 5.2 | 250.5 | 3.5 | 234.6 | 1.8 | 277.5 | 1.2 | 218.3 | 1.7 | 223.2 | 3.0 | 237.4 | 0.3 | 252.9 | 1.6 | 246.2 | 2.1 | 246.8 | 2.5 |
| 8 | 225.3 | 4.6 | 246.0 | 1.3 | 252.2 | 0.0 | 245.0 | 2.8 | 217.9 | 4.8 | 262.4 | 4.3 | 232.3 | 3.3 | 215.8 | 6.0 | 242.1 | 0.2 | 253.6 | 0.6 | 260.0 | 0.4 | 245.3 | 3.3 |
| 9 | 227.5 | 1.8 | 240.4 | 0.3 | 255.3 | 2.6 | 254.3 | 2.5 | 212.2 | 7.2 | 261.8 | 6.9 | 239.0 | 3.5 | 217.9 | 0.3 | 239.9 | 2.3 | 254.3 | 6.9 | 251.4 | 2.6 | 242.4 | 4.3 |
| 10 | 233.6 | 1.6 | 239.5 | 3.5 | 253.6 | 4.9 | 245.1 | 2.1 | 214.1 | 3.6 | 264.1 | 0.9 | 219.9 | 4.0 | 216.4 | 4.6 | 234.8 | 0.6 | 244.1 | 3.7 | 245.9 | 1.7 | 248.3 | 0.9 |
| 11 | 227.4 | 1.8 | 232.6 | 1.0 | 248.9 | 8.1 | 239.0 | 7.1 | 216.6 | 5.8 | 259.3 | 3.2 | 220.0 | 0.4 | 207.0 | 1.6 | 232.9 | 2.2 | 245.6 | 1.0 | 245.8 | 1.1 | 231.9 | 4.5 |
| 12 | 227.8 | 1.4 | 237.1 | 2.0 | 248.1 | 0.5 | 248.9 | 4.4 | 227.8 | 0.1 | 257.3 | 0.1 | 217.4 | 0.8 | 212.4 | 1.8 | 237.8 | 6.5 | 247.8 | 0.1 | 253.6 | 4.7 | 250.9 | 3.3 |
|  |  |  |  |  |  |  |  |  |  |  | **PDI** |  |  |  |  |  |  |  |  |  |  |  |  |  |
| 0 | 0.186 | 0.019 | 0.152 | 0.011 | 0.205 | 0.011 | 0.186 | 0.001 | 0.130 | 0.011 | 0.169 | 0.008 | 0.183 | 0.011 | 0.162 | 0.001 | 0.167 | 0.006 | 0.177 | 0.006 | 0.196 | 0.008 | 0.164 | 0.007 |
| 1 | 0.165 | 0.026 | 0.127 | 0.033 | 0.182 | 0.021 | 0.157 | 0.010 | 0.147 | 0.022 | 0.152 | 0.024 | 0.156 | 0.017 | 0.159 | 0.003 | 0.177 | 0.029 | 0.168 | 0.021 | 0.197 | 0.006 | 0.175 | 0.042 |
| 2 | 0.127 | 0.040 | 0.170 | 0.016 | 0.280 | 0.011 | 0.212 | 0.032 | 0.187 | 0.004 | 0.123 | 0.024 | 0.163 | 0.015 | 0.167 | 0.031 | 0.179 | 0.015 | 0.152 | 0.017 | 0.209 | 0.016 | 0.183 | 0.021 |
| 3 | 0.155 | 0.029 | 0.171 | 0.008 | 0.191 | 0.026 | 0.203 | 0.024 | 0.163 | 0.003 | 0.152 | 0.019 | 0.162 | 0.028 | 0.133 | 0.013 | 0.177 | 0.018 | 0.150 | 0.031 | 0.212 | 0.005 | 0.157 | 0.035 |
| 4 | 0.164 | 0.024 | 0.156 | 0.045 | 0.203 | 0.019 | 0.191 | 0.023 | 0.182 | 0.023 | 0.132 | 0.026 | 0.157 | 0.024 | 0.163 | 0.009 | 0.183 | 0.014 | 0.153 | 0.024 | 0.207 | 0.022 | 0.161 | 0.029 |
| 5 | 0.125 | 0.006 | 0.152 | 0.006 | 0.208 | 0.033 | 0.196 | 0.021 | 0.166 | 0.011 | 0.157 | 0.031 | 0.167 | 0.016 | 0.158 | 0.007 | 0.149 | 0.008 | 0.137 | 0.002 | 0.216 | 0.010 | 0.169 | 0.006 |
| 6 | 0.132 | 0.003 | 0.167 | 0.062 | 0.176 | 0.021 | 0.186 | 0.004 | 0.175 | 0.006 | 0.147 | 0.010 | 0.146 | 0.016 | 0.147 | 0.023 | 0.188 | 0.020 | 0.157 | 0.003 | 0.209 | 0.013 | 0.177 | 0.002 |
| 7 | 0.157 | 0.011 | 0.141 | 0.003 | 0.199 | 0.020 | 0.190 | 0.040 | 0.213 | 0.021 | 0.142 | 0.030 | 0.158 | 0.012 | 0.161 | 0.011 | 0.188 | 0.025 | 0.154 | 0.002 | 0.257 | 0.003 | 0.176 | 0.053 |
| 8 | 0.158 | 0.007 | 0.147 | 0.014 | 0.199 | 0.022 | 0.196 | 0.019 | 0.176 | 0.026 | 0.109 | 0.010 | 0.161 | 0.009 | 0.109 | 0.016 | 0.160 | 0.029 | 0.172 | 0.041 | 0.225 | 0.013 | 0.171 | 0.017 |
| 9 | 0.170 | 0.008 | 0.167 | 0.002 | 0.182 | 0.032 | 0.160 | 0.004 | 0.221 | 0.002 | 0.147 | 0.013 | 0.215 | 0.012 | 0.111 | 0.042 | 0.150 | 0.006 | 0.156 | 0.001 | 0.220 | 0.006 | 0.162 | 0.016 |
| 10 | 0.131 | 0.001 | 0.172 | 0.002 | 0.182 | 0.001 | 0.187 | 0.027 | 0.157 | 0.015 | 0.104 | 0.016 | 0.132 | 0.019 | 0.122 | 0.043 | 0.150 | 0.045 | 0.139 | 0.026 | 0.184 | 0.018 | 0.176 | 0.016 |
| 11 | 0.178 | 0.001 | 0.147 | 0.030 | 0.220 | 0.016 | 0.184 | 0.003 | 0.173 | 0.003 | 0.133 | 0.048 | 0.164 | 0.001 | 0.168 | 0.004 | 0.177 | 0.040 | 0.163 | 0.038 | 0.206 | 0.015 | 0.161 | 0.019 |
| 12 | 0.171 | 0.022 | 0.161 | 0.028 | 0.213 | 0.017 | 0.197 | 0.012 | 0.166 | 0.017 | 0.134 | 0.009 | 0.175 | 0.015 | 0.143 | 0.023 | 0.166 | 0.019 | 0.130 | 0.036 | 0.221 | 0.002 | 0.195 | 0.011 |
| **Zeta Potential (mV)** | | | | | | | | | | | | | | | | | | | | | | | | |
| 0 | -28.3 | 0.2 | -34.0 | 0.9 | -30.5 | 0.5 | -44.1 | 0.6 | -27.7 | 2.8 | -42.9 | 0.3 | -29.6 | 0.1 | -41.3 | 0.4 | -28.1 | 0.9 | -41.3 | 0.6 | -29.5 | 0.4 | -42.5 | 0.9 |
| 1 | -26.6 | 0.1 | -35.7 | 0.3 | -29.8 | 0.5 | -42.5 | 1.0 | -28.0 | 0.9 | -39.2 | 0.1 | -28.8 | 0.8 | -46.5 | 0.2 | -28.4 | 0.3 | -41.3 | 0.4 | -29.2 | 1.2 | -43.9 | 0.9 |
| 2 | -28.1 | 0.3 | -34.3 | 0.7 | -30.6 | 1.0 | -41.4 | 0.1 | -30.6 | 0.7 | -41.0 | 0.6 | -28.5 | 0.6 | -36.5 | 0.5 | -29.6 | 0.5 | -40.0 | 0.7 | -28.2 | 1.6 | -40.9 | 0.4 |
| 3 | -31.7 | 0.8 | -35.0 | 0.9 | -35.5 | 0.3 | -44.0 | 0.7 | -34.9 | 0.6 | -42.1 | 0.2 | -34.1 | 0.9 | -35.7 | 0.8 | -30.7 | 0.8 | -40.6 | 0.3 | -35.0 | 0.4 | -44.6 | 0.5 |
| 4 | -29.5 | 0.6 | -33.3 | 0.9 | -34.2 | 0.5 | -40.7 | 0.4 | -34.4 | 1.0 | -41.3 | 1.6 | -30.0 | 1.2 | -42.5 | 1.1 | -33.5 | 0.6 | -45.9 | 0.7 | -33.5 | 0.8 | -41.5 | 0.4 |
| 5 | -20.2 | 0.0 | -38.6 | 1.1 | -35.3 | 1.3 | -41.3 | 1.8 | -30.6 | 0.8 | -41.0 | 0.1 | -30.6 | 0.4 | -44.5 | 0.5 | -34.2 | 0.1 | -44.2 | 0.3 | -34.3 | 0.2 | -45.0 | 0.7 |
| 6 | -24.8 | 0.3 | -36.9 | 0.3 | -32.5 | 0.8 | -45.0 | 0.7 | -28.9 | 1.3 | -43.9 | 0.7 | -30.1 | 0.0 | -42.8 | 0.3 | -30.9 | 0.4 | -44.6 | 0.4 | -32.7 | 1.3 | -47.5 | 0.9 |
| 7 | -28.0 | 0.1 | -38.5 | 0.4 | -35.7 | 0.4 | -42.3 | 1.8 | -31.1 | 0.1 | -42.2 | 0.8 | -33.8 | 0.7 | -33.0 | 0.1 | -34.2 | 0.6 | -44.0 | 0.2 | -33.2 | 0.7 | -43.3 | 0.8 |
| 8 | -29.8 | 1.3 | -36.0 | 0.2 | -29.1 | 0.8 | -41.5 | 0.3 | -26.2 | 0.1 | -45.0 | 0.4 | -29.1 | 0.1 | -40.0 | 0.4 | -28.5 | 0.6 | -43.3 | 1.0 | -29.1 | 0.1 | -45.0 | 0.4 |
| 9 | -30.6 | 0.0 | -37.5 | 0.0 | -35.8 | 0.4 | -44.0 | 0.3 | -34.0 | 0.1 | -44.8 | 1.1 | -33.4 | 0.3 | -44.6 | 1.7 | -34.7 | 0.2 | -45.1 | 0.3 | -34.8 | 0.7 | -43.3 | 1.4 |
| 10 | -25.0 | 0.2 | -34.3 | 0.6 | -30.6 | 0.8 | -43.2 | 0.1 | -27.5 | 0.2 | -38.9 | 1.0 | -29.5 | 1.2 | -41.7 | 1.1 | -30.3 | 0.7 | -43.3 | 0.1 | -29.9 | 1.3 | -46.3 | 0.6 |
| 11 | -32.5 | 0.4 | -35.0 | 0.8 | -38.0 | 0.6 | -40.2 | 0.4 | -32.0 | 0.9 | -44.3 | 0.7 | -35.4 | 1.7 | -40.3 | 0.0 | -30.1 | 0.4 | -39.5 | 0.2 | -27.4 | 0.4 | -42.4 | 0.6 |
| 12 | -27.0 | 0.2 | -32.6 | 0.7 | -32.4 | 0.7 | -44.2 | 0.4 | -28.1 | 1.1 | -43.8 | 1.3 | -31.8 | 0.1 | -43.2 | 0.5 | -29.5 | 0.2 | -42.7 | 0.5 | -28.0 | 1.0 | -43.7 | 0.3 |

**Figure S1**. Response Surfaces obtained with the Design Expert software, for: A) Particle size; B) PDI and C) Zeta potential for NLCs formulations composed of cetyl palmitate (CP), Dhaykol 6040 (DK) and Pluronic F68 (P68). Graphics created using Design-Expert software, version 10 (www.statease.com).

**Figure S2**. Response Surfaces obtained with the Design Expert software, for: A) Particle size; B) PDI and C) Zeta potential for NLCs formulations composed of cetyl palmitate (CP), Dhaykol 6040 (DK) and Tween 80 (T80). Graphics created using Design-Expert software, version 10 (www.statease.com).

**Figure S3**. Response Surfaces obtained with the Design Expert software, for: A) Particle size; B) PDI and C) Zeta potential for NLCs formulations composed of avocado butter (AB), copaiba oil (CO) and Pluronic F68 (P68). Graphics created using Design-Expert software, version 10 (www.statease.com).

**Figure S4**. Response Surfaces obtained with the Design Expert software, for: A) Particle size; B) PDI and C) Zeta potential for NLCs formulations composed of avocado butter (AB), copaiba oil (CO) and Tween 80 (T80). Graphics created using Design-Expert software, version 10 (www.statease.com).

**Figure S5**. Response Surfaces obtained with the Design Expert software, for: A) Particle size; B) PDI and C) Zeta potential for NLCs formulations composed of avocado butter (AB), olive oil (OO) and Pluronic F-68 (P68). Graphics created using Design-Expert software, version 10 (www.statease.com).

**Figure S6**. Response Surfaces obtained with the Design Expert software, for: A) Particle size; B) PDI and C) Zeta potential for NLCs formulations composed of avocado butter (AB), olive oil (OO) and Tween 80 (T80). Graphics created using Design-Expert software, version 10 (www.statease.com).

**Figure S7**. Desirability graphs obtained with the Design Expert software: A) NLC-CP1; B) NLC-CP2; C) NLC-CO1; D) NLC-CO2; E) NLC-OO1 and F) NLC-OO2. See table 1 for abbreviation and NLC composition. Desirable criteria for optimization: 1) Smaller particle sizes; 2) PDI < to 0.25 and 3) Zeta potential > 20, in modulus. Graphics created using Design-Expert software, version 10 (www.statease.com).

**
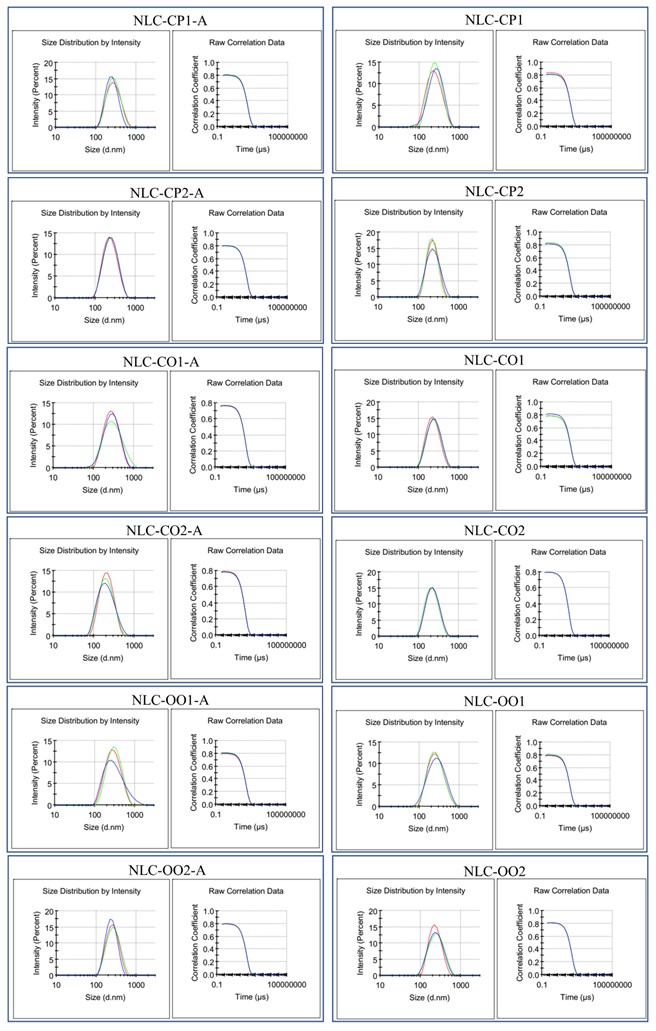
**

**Figure S8.** DLS plots of size distribution (measured by intensity of scattered light) and data correlation for the six optimized NLC formulations (see Table 4) and their controls. Graphs obtained from zetasizer software, version 7.13 (www.malvernpanalytical.com)

**Figure S9.** TEM micrographs of the optimized nanostructured lipid carriers containing articaine. Magnification: 27800x (1 and 2), 35970x (4), 46460x (3 and 5) and 77500 (6). The images were edited with ImageJ software v.1.52a (https://imagej.nih.gov/ij/).


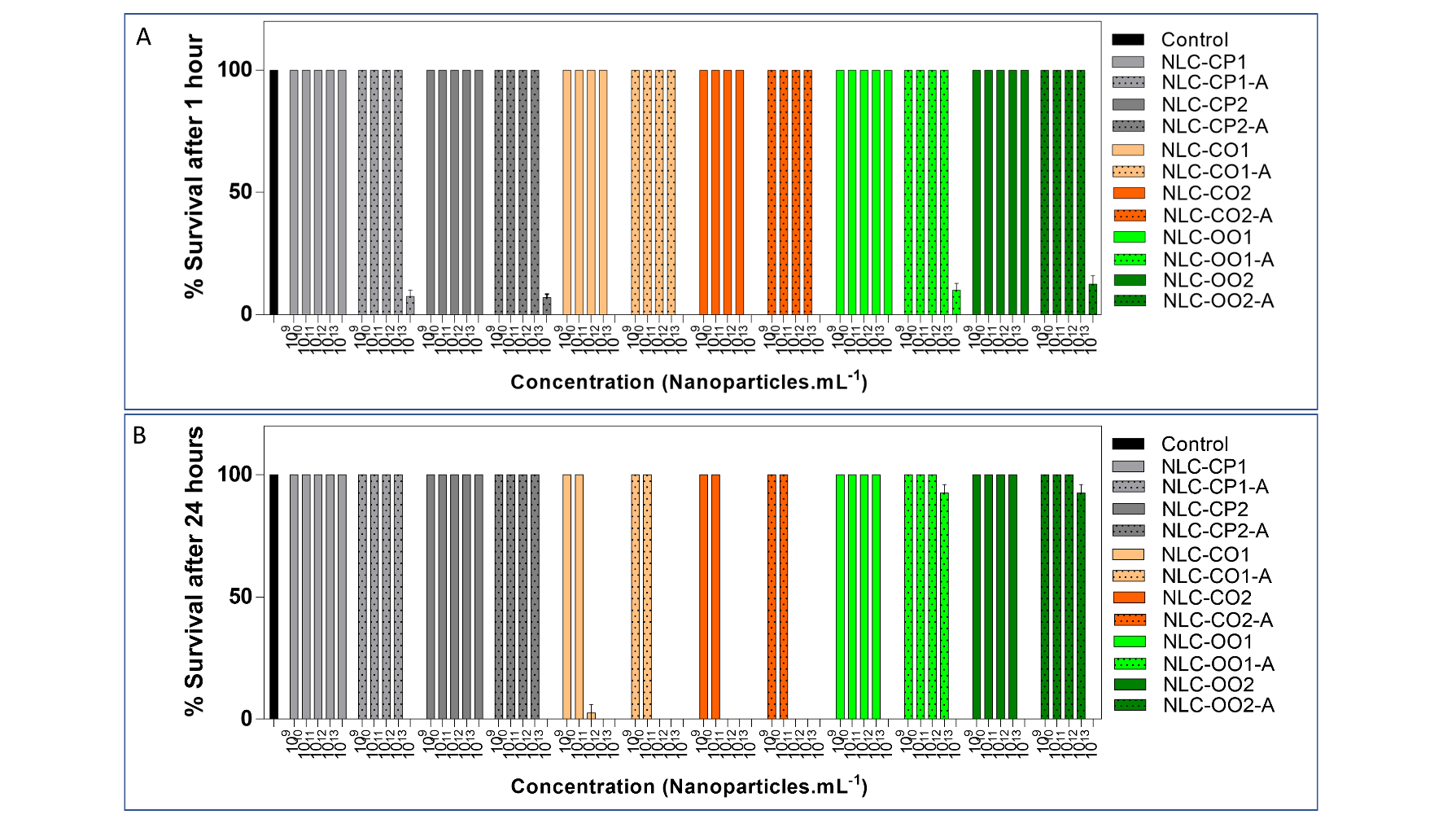


**Figure S10**. Zebrafish larvae survival (%) after 1 (A) and 24 (B) hours post incubation with the optimized NLC formulations, at different concentrations.


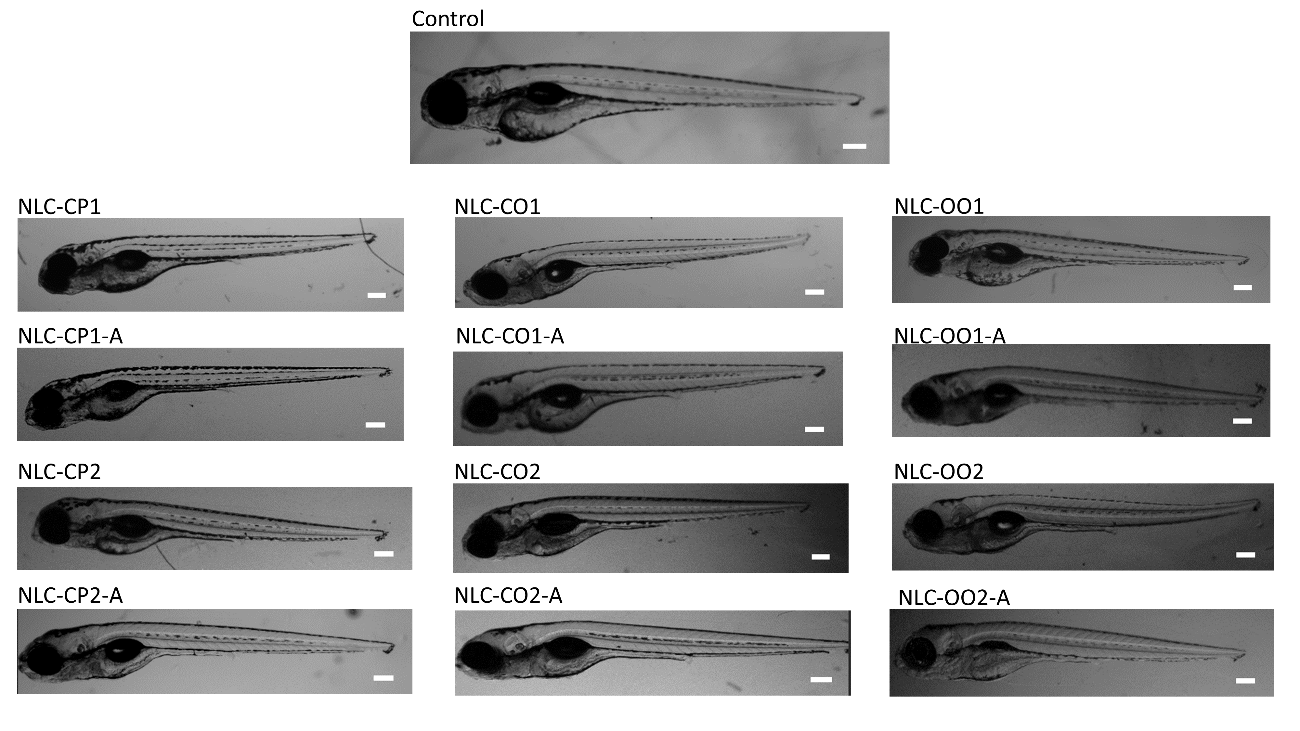


**Figure S11**. Lateral view of larvae after 1 hour post incubation with the optimized NLC formulations at the concentration of 10^12^ particles/mL. Scale bar = 200 µm. The images were edited with ImageJ software v.1.52a (https://imagej.nih.gov/ij/).


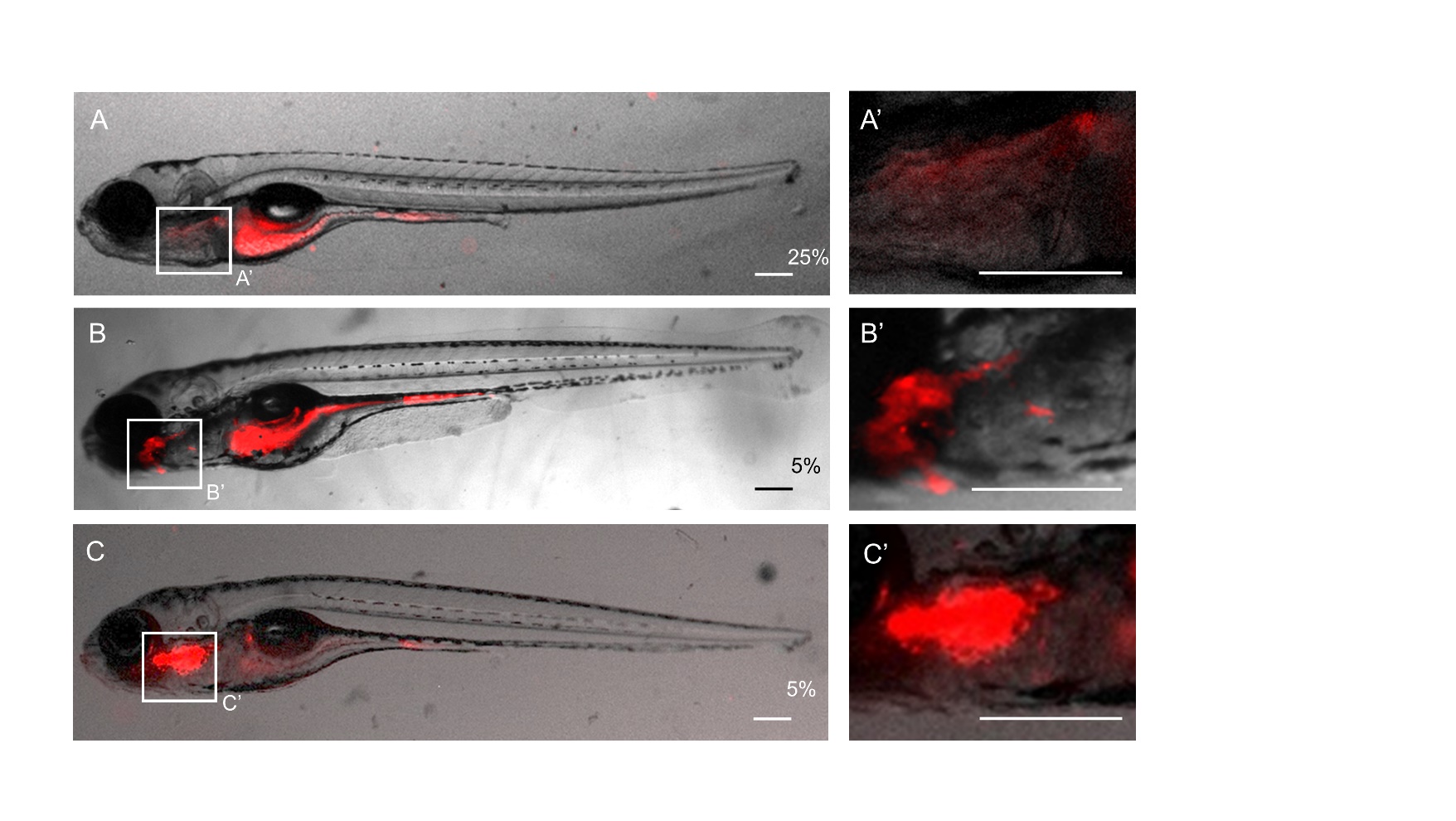


**Figure S12.** Lateral view of larvae after 24 hours post incubation with rhodamine-labeled NLC. A, B, C) Representative images of larvae with NLC-rhodamine in the GI tract and esophagus area. A’, B' C’) highlight the presence of NLC-rhodamine in the esophagus area. Scale bar = 200 μm. The images (A, C-H) were edited using ImageJ software v.1.52a (https://imagej.nih.gov/ij/).

**Figure S13.** Stability tests performed with the optimized NLC formulations and their controls. Changes in size (A, D, G), PDI (B, E, H) and zeta potential (C, F, I) over one year of storage at room temperature.
